# Dietary Bisphenol A Exposure and Urinary Bisphenol A Levels During Late Pregnancy: Associations with Inflammatory and Oxidative Stress Biomarkers

**DOI:** 10.3390/nu18172836

**Published:** 2026-08-28

**Authors:** Sümeyye Begüm Atalan, Aylin Ayaz

**Affiliations:** 1 Department of Nutrition and Dietetics, Faculty of Health Sciences, Kastamonu University, Kastamonu 37150, Türkiye; 2 Department of Nutrition and Dietetics, Faculty of Health Sciences, Hacettepe University, Ankara 06100, Türkiye

**Keywords:** bisphenol A, pregnancy, oxidative stress, inflammation, dietary inflammatory index, dietary total antioxidant capacity, dietary exposure, 8-OHdG, 8-isoprostane, fetal outcomes, endocrine-disrupting chemicals

## Abstract

**Background/Objectives:** Pregnant women are vulnerable to endocrine-disrupting chemicals such as bisphenol A (BPA). However, associations of maternal BPA exposure with oxidative stress, inflammation, the Dietary Inflammatory Index (DII), dietary total antioxidant capacity (DTAC), and fetal/neonatal outcomes remain unclear. This study evaluated urinary and estimated dietary BPA exposure in conjunction with DII and DTAC during late pregnancy and examined their associations with maternal oxidative stress and inflammatory biomarkers and fetal/neonatal outcomes. **Methods:** This cross-sectional study included 86 third-trimester pregnant women at a Turkish tertiary hospital. BPA exposure was assessed using creatinine-adjusted urinary BPA concentrations and dietary intake estimated from 3-day dietary records and a food frequency questionnaire. Urinary and serum biomarkers were measured, and DII and DTAC scores calculated. Analyses included correlation, multivariable regression, and mediation. **Results:** Creatinine-adjusted urinary BPA correlated strongly with urinary 8-isoprostane (*r* = 0.694, *p* < 0.001) and 8-hydroxy-2′-deoxyguanosine (8-OHdG; *r* = 0.880, *p* < 0.001), but not after multivariable adjustment. Neither urinary nor dietary BPA exposure showed consistent adjusted associations with serum oxidative stress or inflammatory biomarkers or significant associations with fetal/neonatal outcomes. DII and DTAC correlated with selected inflammatory biomarkers, particularly TNF-α. Dietary BPA hazard quotients exceeded 1 using EFSA’s updated 2023 tolerable daily intake, suggesting a potential health concern. **Conclusions:** Urinary BPA correlated strongly with urinary oxidative damage biomarkers, but not after multivariable adjustment. Neither urinary nor dietary BPA showed consistent adjusted associations with maternal oxidative stress or inflammatory biomarkers or significant associations with fetal/neonatal outcomes. DII and DTAC may be related to inflammatory biomarker variability in late pregnancy.

## 1. Introduction

Endocrine-disrupting chemicals (EDCs) are exogenous compounds that can interfere with hormonal systems and may affect developmental processes, particularly during sensitive periods such as pregnancy [1,2]. Among these compounds, bisphenol A (BPA) is one of the most extensively studied EDCs in the literature due to its widespread use, ubiquitous exposure, and potential adverse health effects [3,4,5].

BPA is widely used in the production of polycarbonate plastics and epoxy resins and may migrate into foods through food contact packaging materials [6,7]. Although BPA exposure can occur through multiple routes, the gastrointestinal tract is considered the predominant route in the general population, with dietary intake accounting for a substantial proportion of total exposure [8,9].

Food packaging materials and canned products are major sources of BPA exposure, and several studies have shown that higher consumption of canned foods is associated with increased BPA levels [10,11]. In its most recent assessment, the European Food Safety Authority (EFSA) substantially reduced the tolerable daily intake for BPA to 0.2 ng/kg/day [12]. Epidemiological and experimental studies suggest that BPA exposure may be associated with metabolic disturbances and adverse developmental outcomes [3,13]. BPA has also been shown to cross the placenta from the maternal circulation into the fetal circulation and has been detected in various biological samples from mother–infant pairs, including serum, urine, placenta, and cord blood [14]. Therefore, the effects of prenatal BPA exposure on fetal growth and birth outcomes have been increasingly investigated. However, the direction and strength of the associations between BPA levels and outcomes such as birth weight, fetal biometric measurements, and gestational duration remain inconsistent across studies [15,16,17].

One proposed mechanism underlying the biological effects of BPA involves oxidative stress and inflammation. Both experimental and human studies have demonstrated that BPA exposure may increase the production of reactive oxygen species (ROS), impair antioxidant defense systems, and trigger inflammatory responses [18,19,20]. Oxidative stress is characterized by an imbalance between ROS production and antioxidant defense systems, leading to oxidative damage in cellular components such as proteins, lipids, and DNA [21].

Although increased oxidative stress during pregnancy is considered a physiological process, excessive oxidative stress has been associated with impaired placental development and function, as well as adverse pregnancy outcomes, including low birth weight, preterm birth, and fetal growth restriction [22,23]. In this context, oxidative stress and inflammatory pathways have been proposed as potential mechanisms underlying the biological effects of BPA exposure. However, the associations between BPA exposure and oxidative stress and inflammatory biomarkers remain inconsistent in the literature, with findings varying across studies [18,24].

In addition, dietary components are increasingly recognized as important determinants of inflammation and oxidative stress [25,26]. Dietary patterns rich in antioxidants have been shown to reduce ROS production, thereby limiting oxidative damage and suppressing inflammatory responses [27]. In contrast, proinflammatory dietary patterns have been shown to enhance inflammatory responses in experimental studies [28,29].

The Dietary Inflammatory Index (DII) was developed to assess the inflammatory potential of the diet [30,31], and higher DII scores have been associated with increased inflammation and may be linked to adverse birth outcomes during pregnancy [32,33]. Similarly, a higher dietary inflammatory potential during pregnancy has been associated with low birth weight, small-for-gestational-age birth, and impaired fetal growth [34,35,36].

Dietary total antioxidant capacity (DTAC) reflects the overall potential of the diet to counteract oxidative stress, and dietary patterns rich in antioxidant components have been shown to reduce ROS production, thereby limiting oxidative damage and suppressing inflammatory responses [27]. Diets with higher antioxidant capacity during pregnancy have been reported to be associated with maternal oxidative stress levels and birth outcomes [37,38]. Because DTAC reflects the overall dietary pattern rather than individual nutrients alone, it is considered an important indicator for evaluating biological processes related to oxidative stress [39,40]. Although it has been proposed that the inflammatory potential and antioxidant capacity of the diet may modify the biological effects of environmental exposures, the role of these interactions, particularly in relation to pregnancy outcomes, remains unclear, and current evidence suggests that these relationships are complex and multifactorial [41,42]. While associations between BPA exposure and oxidative stress and inflammatory biomarkers have been extensively investigated [43,44], holistic approaches simultaneously evaluating dietary BPA intake together with dietary inflammatory and antioxidant characteristics remain limited.

Accordingly, this descriptive cross-sectional study aimed to evaluate maternal urinary and estimated dietary BPA exposure in conjunction with the Dietary Inflammatory Index and dietary total antioxidant capacity during late pregnancy and to examine their associations with maternal oxidative stress and inflammatory biomarkers and fetal and neonatal outcomes.

## 2. Materials and Methods

### 2.1. Study Design

This cross-sectional, descriptive study was conducted at a single center in Kastamonu, Türkiye.

**Figure 1:**
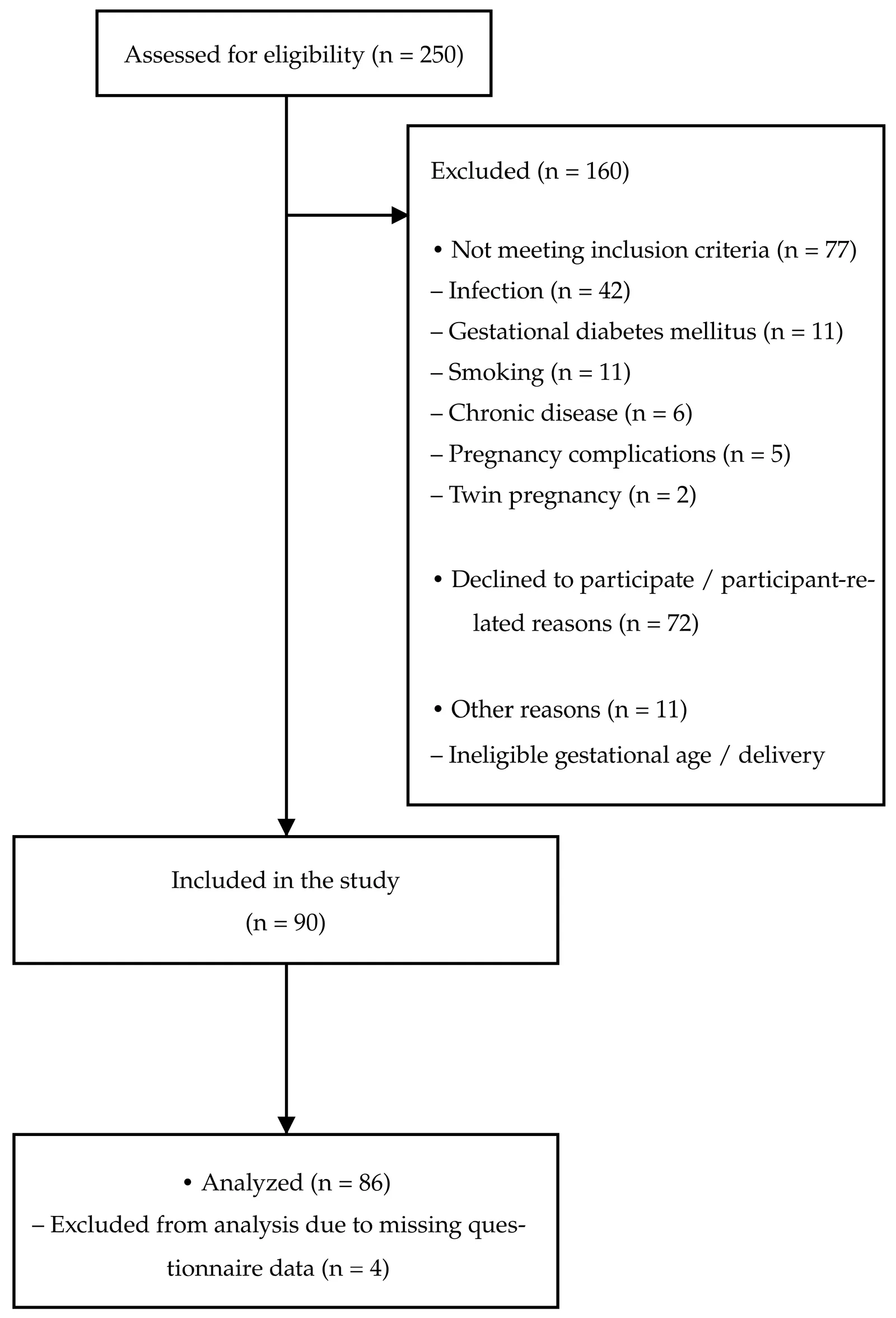
Diagram of participant selection, including reasons for exclusion and missing data.

The required sample size was determined using an a priori power analysis performed with G*Power (version 3.1.9.2). The sample size calculation was based on a correlation effect size of *r* = 0.316, using a two-sided test. With a significance level of α = 0.05 and a statistical power of 80%, the minimum required sample size was 73 participants. To account for potential missing or incomplete data, the target sample size was set at 87 participants. The final study sample comprised 86 participants. Among 250 pregnant women assessed for eligibility during the study period, 90 met the inclusion and exclusion criteria and were enrolled. Four participants with incomplete questionnaire data were excluded from the analyses, leaving 86 pregnant women in the final study sample. The participant selection process is shown in the flow diagram (see Figure 1).

### 2.2. Participants

This study was conducted at Kastamonu Training and Research Hospital between October 2023 and August 2024. The study population consisted of healthy pregnant women aged 19–40 years in the third trimester (27–36 weeks of gestation).

Women were included if they had no physician-diagnosed chronic disease, were within the eligible gestational age range, agreed to participate voluntarily, and provided written informed consent. Participants were excluded if they had a chronic disease, acute infection, a history of COVID-19 within the previous three months, an autoimmune disease or malignancy, communication difficulties or sensory impairments, a multiple pregnancy, pregnancy-related complications such as gestational diabetes mellitus, preeclampsia, or gestational hypertension, chronic alcohol use or smoking, occupational chemical exposure, or severe psychiatric disorders (see Figure 1).

The study protocol was approved by the Kastamonu University Clinical Research Ethics Committee (Decision No: 2023-KAEK-85; Date: 2 August 2023). Written informed consent was obtained from all participants before enrollment. The study was conducted in accordance with the principles of the Declaration of Helsinki.

### 2.3. Data Collection and Dietary Assessment

Data were collected through face-to-face interviews using a structured questionnaire. The questionnaire included items on participants’ sociodemographic characteristics, anthropometric measurements, pregnancy history, and dietary habits.

Dietary intake was assessed using three-day retrospective food consumption records, covering two weekdays and one weekend day, together with a semi-quantitative food frequency questionnaire that covered the previous three months [45]. A food photograph atlas was used to assist participants in accurately identifying foods and beverages and estimating portion sizes [46]. Average daily food consumption was calculated from the reported consumption frequencies and quantities.

Dietary intake data were analyzed using the Computer-Aided Nutrition Information System (BeBiS) software, version 9, to determine daily energy, macronutrient, and micronutrient intakes [47]. For flavonoids and isoflavones not available in the software database, data from the United States Department of Agriculture (USDA) food composition databases were used and incorporated into the analyses [48,49].

Using the dietary data obtained, participants’ daily energy and nutrient intakes were calculated. Dietary BPA exposure was estimated using both the 3-day dietary records and the FFQ, whereas DII and DTAC were derived from FFQ-based energy and nutrient intake data.

### 2.4. Dietary Inflammatory Index and Dietary Total Antioxidant Capacity

The inflammatory potential of the diet was evaluated using the DII developed by Shivappa et al. (2014) [31]. To calculate DII scores, nutrient intakes obtained from the food frequency questionnaire were standardized using global reference values. For each dietary component, Z-scores were calculated, converted into percentile scores, and then centered percentile scores. These scores were multiplied by the corresponding inflammatory effect scores assigned to each dietary component, and the sum of all component scores was used to calculate individual DII scores.

A total of 43 dietary components were included in the DII calculation in the present study. Trans fatty acids were excluded because daily intake could not be estimated, and alcohol intake was not considered an appropriate parameter due to the exclusion criteria and the participants’ pregnancy status. Higher DII scores indicate a more proinflammatory dietary pattern, whereas lower scores reflect a more anti-inflammatory dietary pattern [31].

Dietary total antioxidant capacity (DTAC) was calculated using food frequency questionnaire data. The antioxidant capacities of foods were determined using the database developed by Carlsen et al. (2010) based on the Ferric Reducing Antioxidant Power (FRAP) method [50]. DTAC was calculated by multiplying the average daily consumption of each food item by its antioxidant capacity value and summing the resulting values for each participant. To account for interindividual variation in energy intake, DTAC values were standardized per 1000 kcal of energy intake.

### 2.5. Calculation of Dietary BPA Exposure

To estimate dietary BPA exposure, foods consumed by the participants were classified into eight main groups according to shared characteristics. These groups included milk and dairy products; meat and meat products (including eggs, legumes, oilseeds, fish, and meat); vegetables and fruits; bread and cereals; fats and oils; sweets; non-alcoholic beverages (excluding water); and other foods (including canned products, tomato paste, sauces, and chips) [51].

Dietary BPA exposure was calculated using two different dietary assessment methods. In the first method, data obtained from three-day food consumption records were used, whereas in the second method, data from the food frequency questionnaire (FFQ), reflecting participants’ habitual dietary patterns over the previous three months, were utilized [52,53]. To determine BPA concentrations in foods, middle bound values reported by the EFSA for canned and non-canned products were used [9]. In addition, BPA concentrations for canned foods were defined based on data from a study investigating BPA migration from canned foods and beverages marketed in Türkiye [54].

Daily dietary BPA exposure was calculated by multiplying the BPA concentration assigned to each food group by the corresponding amount consumed. The resulting value was then divided by participants’ body weight [52]. Accordingly, the estimated daily intake (EDI) of BPA (ng/kg body weight/day) was calculated using the following formula:

$$EDI=\sum_{j=1}^{n}\frac{C_{j}\;\times \;{IR}_{j}\;}{BW}$$

where *C_j_* (ng/g) denotes the BPA concentration in foods, *IR__j__* (g/day) denotes the individual daily food consumption amount of each food, BW (kg) denotes body weight, *n* is the total number of food items included in the assessment, and *j* represents the index of each food item (*j* = 1, 2,…, *n*).

To evaluate the risk level associated with estimated dietary BPA exposure, the hazard quotient (HQ) was calculated. The HQ value was calculated by dividing the EDI for BPA by the tolerable daily intake (TDI). In the present study, HQ calculations were performed using two TDI values established by EFSA. Accordingly, separate HQ values were calculated based on the previous EFSA TDI from 2015 and the updated EFSA TDI from 2023. An HQ value greater than 1 was considered indicative of a potential health risk [9,12].

### 2.6. Anthropometric Measurements and Clinical Data

Participants’ body weight was measured to the nearest 0.05 kg using a digital scale while wearing light clothing and no shoes, and height was measured using a stadiometer with participants standing upright in the Frankfurt plane position [55]. Current body weight during pregnancy was recorded. Pre-pregnancy body weight was self-reported, pre-pregnancy body mass index (BMI) was calculated as weight (kg)/height (m^2^), and BMI was classified according to the World Health Organization (WHO) criteria [56].

Estimated fetal weight (EFW) values were obtained from routine measurements performed during outpatient follow-up visits and were retrieved from participants’ medical records at the time of study enrollment. Neonatal anthropometric measurements obtained routinely after delivery, including birth weight, birth length, and head circumference, were also retrieved from medical records.

### 2.7. Collection of Biological Samples and Laboratory Analyses

#### 2.7.1. Urinary BPA and Creatinine Analyses

For BPA analyses, first-morning urine samples were collected from the participants using de-plasticized glass urine containers to standardize the timing of sample collection and minimize variation in urine dilution. To minimize potential BPA contamination during sample collection, transportation, and storage, contact with plastic materials was avoided throughout the procedure. After collection, the urine samples were covered with aluminum foil, transferred into de-plasticized amber glass bottles, wrapped again in aluminum foil, and stored at −20 °C until analysis.

Total urinary BPA concentrations were determined using a modified extraction and high-performance liquid chromatography (HPLC) method originally described by Yang et al. (2003) and subsequently applied to urine samples by Celik et al. (2025) [57,58]. The BPA analytical standard was obtained from Sigma-Aldrich (St. Louis, MO, USA), and glucuronidase/arylsulfatase from *Helix pomatia* was obtained from Roche (Mannheim, Germany).

For sample preparation, 500 µL of urine was combined with 30 µL of 2.0 M ammonium acetate buffer (pH 5.0) and 10 µL of glucuronidase/arylsulfatase. After vortex mixing, the mixture was incubated in a water bath at 37 °C for 3 h to hydrolyze conjugated BPA. BPA was then extracted using 5 mL of an n-hexane–diethyl ether mixture (70:30, *v*/*v*). Following centrifugation at 3500 rpm for 5 min, 3 mL of the organic supernatant was transferred into a glass tube and evaporated under a stream of nitrogen. The dried residues were stored at −20 °C until chromatographic analysis.

On the day of analysis, the residues were reconstituted in 300 µL of 60% acetonitrile, and a 100 µL aliquot was injected into an Agilent 1200 Series HPLC system (Agilent Technologies, Santa Clara, CA, USA). Chromatographic separation was performed using a Spherisorb C18 ODS2 column (25 cm × 4.6 mm internal diameter; 5 µm particle size) maintained at 25 °C. The mobile phase consisted of acetonitrile and 2.5% aqueous tetrahydrofuran and was delivered at a flow rate of 0.4 mL/min. Gradient elution from 60:40 to 5:95 was applied over a total run time of 40 min. BPA was measured using fluorescence detection at excitation and emission wavelengths of 230 and 315 nm, respectively. The retention time of BPA in the urine samples was approximately 18.5–18.6 min.

Calibration standards were prepared at BPA concentrations of 2.5, 5, 10, 25, 50, 100, 500, and 1000 ng/mL. The method-specific limit of detection (LOD) and limit of quantification (LOQ) were 1.0 and 2.5 ng/mL, respectively. All urinary BPA samples were analyzed in duplicate.

Urinary creatinine was measured using an HPLC procedure originally described by Jen et al. (2002) and subsequently applied with modifications by Celik et al. (2025) [58,59]. Before chromatographic analysis, the urine samples were diluted 1:1000 with deionized water. Separation was performed on a C18 column using 15 mM potassium dihydrogen phosphate buffer containing 2.5% methanol (pH 7.0) as the mobile phase. Creatinine was quantified using a UV detector at 235 nm, and the resulting concentrations were corrected for the dilution introduced during sample preparation. Urinary BPA concentrations were subsequently normalized to urinary creatinine concentrations and expressed as µg/g creatinine.

#### 2.7.2. Urinary Oxidative Stress Biomarkers

A separate aliquot of each first-morning urine sample was stored at −80 °C until the analysis of urinary 8-hydroxy-2′-deoxyguanosine (8-OHdG) and 8-isoprostane.

Urinary 8-isoprostane was measured in duplicate using a commercially available competitive ELISA kit (BT LAB, Shanghai Korain Biotech Co., Ltd., Shanghai, China; catalog no. EA0077Hu) according to the manufacturer’s instructions. Absorbance was measured at 450 nm using a BioTek ELx800 microplate reader (BioTek Instruments, Inc., Winooski, VT, USA), and washing steps were performed using a BioTek ELx50 automated strip washer (BioTek Instruments, Inc., Winooski, VT, USA). The manufacturer-reported intra-assay and inter-assay coefficients of variation were <10% and <12%, respectively, and the assay sensitivity was 14.27 ng/L.

Urinary 8-OHdG was measured in duplicate using a competitive ELISA kit (Elabscience Biotechnology Inc., Houston, TX, USA; catalog no. E-EL-0028) according to the manufacturer’s instructions. Absorbance was measured at 450 ± 2 nm using the BioTek ELx800 microplate reader, and washing steps were performed using the BioTek ELx50 automated strip washer. For the 8-OHdG assay, the manufacturer-reported intra-assay and inter-assay coefficients of variation were <7% and <9%, respectively, and the assay sensitivity was 0.94 ng/mL.

#### 2.7.3. Serum Inflammatory and Oxidative Stress Biomarkers

To determine serum inflammatory biomarkers, oxidative status parameters, antioxidant enzyme activities, and malondialdehyde (MDA), venous blood samples were collected from participants after an overnight fast of at least 8 h. The blood samples were centrifuged at 3000 rpm for 10 min to separate the serum, which was then stored at −80 °C until analysis.

Serum interleukin-1 beta (IL-1β), interleukin-6 (IL-6), interleukin-10 (IL-10), and tumor necrosis factor-alpha (TNF-α) concentrations were measured in duplicate using commercially available ELISA kits (BT LAB, China; catalog nos. E0143Hu, E0090Hu, E0102Hu, and E0082Hu, respectively) according to the manufacturer’s instructions. Absorbance was measured at 450 nm using a BioTek ELx800 microplate reader, and washing steps were performed using a BioTek ELx50 automated strip washer. The manufacturer-reported assay sensitivities were 10.07 pg/mL for IL-1β, 1.03 ng/L for IL-6, 2.59 pg/mL for IL-10, and 1.52 ng/L for TNF-α. The manufacturer-reported intra-assay and inter-assay coefficients of variation were <8% and <10%, respectively.

Serum total antioxidant status (TAS) was measured using a commercially available colorimetric kit (Rel Assay Diagnostics, Gaziantep, Türkiye;catalog no. RL0017) on a Mindray BS-400 fully automated biochemical analyzer (Shenzhen Mindray Bio-Medical Electronics Co., Ltd., Shenzhen, China). The method was based on the ability of antioxidants in the sample to suppress the characteristic color of the 2,2′-azino-bis(3-ethylbenzothiazoline-6-sulfonic acid) radical cation. TAS results were expressed as mmol Trolox equivalent/L.

Serum total oxidant status (TOS) was measured using a commercially available colorimetric kit (Rel Assay Diagnostics; catalog no. RL0024) on the Mindray BS-400 analyzer. In this method, oxidants in the sample oxidized the ferrous ion–o-dianisidine complex to ferric ions. The ferric ions subsequently formed a colored complex with xylenol orange in an acidic medium, and the color intensity was proportional to the total amount of oxidant molecules in the sample. The assay was calibrated using hydrogen peroxide, and the results were expressed as µmol H_2_O_2_ equivalent/L.

After converting TAS values from mmol Trolox equivalent/L to µmol Trolox equivalent/L, the oxidative stress index (OSI) was calculated as follows:

OSI (arbitrary units) = [TOS (µmol H_2_O_2_ equivalent/L)/TAS (µmol Trolox equivalent/L)] × 100.

Serum superoxide dismutase (SOD) activity was measured using a colorimetric kit (Otto Scientific, Ankara, Türkiye; catalog no. Otto3047) on the Mindray BS-400 analyzer. The method used xanthine and xanthine oxidase to generate superoxide radicals, which reacted with 2-(4-iodophenyl)-3-(4-nitrophenol)-5-phenyltetrazolium chloride to form a red formazan dye. SOD activity was determined according to the degree of inhibition of this reaction.

Serum catalase activity was measured colorimetrically using a commercial kit (Elabscience; catalog no. E-BC-K031-S). The decomposition of hydrogen peroxide by catalase was stopped using ammonium molybdate. Residual hydrogen peroxide reacted with ammonium molybdate to form a yellow complex, and absorbance was measured at 405 nm using a microplate reader (Rel Assay Diagnostics, Gaziantep, Türkiye; model RL0505).

Serum MDA levels were determined using a thiobarbituric acid-based colorimetric method (Otto Scientific; catalog no. Otto1001). The serum sample was mixed with two volumes of cold 10% (*w*/*v*) trichloroacetic acid to precipitate proteins. After centrifugation, an aliquot of the supernatant was mixed with an equal volume of 0.67% (*w*/*v*) thiobarbituric acid and incubated in a boiling water bath for 10 min. After cooling, the absorbance of the resulting colored product was measured at 532 nm using the Rel Assay Diagnostics RL0505 microplate reader.

Serum glutathione peroxidase (GPx) activity was measured using a colorimetric kit (Otto Scientific; catalog no. Otto2085) on the Mindray BS-400 analyzer. The method was based on the GPx-catalyzed oxidation of glutathione by cumene hydroperoxide. Oxidized glutathione was subsequently converted back to its reduced form, accompanied by the oxidation of NADPH to NADP^+^. GPx activity was determined by measuring the decrease in absorbance at 340 nm.

Serum C-reactive protein (CRP) was measured using an immunoturbidimetric assay (Otto Scientific; catalog no. OttoBC138) on the Mindray BS-400 analyzer. Anti-CRP antibodies reacted with CRP in the serum samples to form antigen–antibody complexes, and the resulting agglutination was measured turbidimetrically.

### 2.8. Statistical Analysis

Statistical analyses were performed using IBM SPSS Statistics version 23.0 (IBM Corp., Armonk, NY, USA), R software (version 4.4.1), and SmartPLS software (version 4.0). The normality of continuous variables was assessed using the Kolmogorov–Smirnov test. Continuous variables were presented as mean ± standard deviation or median (Q1–Q3), whereas categorical variables were expressed as frequencies and percentages. For comparisons between groups, independent samples *t*-tests and one-way analysis of variance (ANOVA) were used for normally distributed variables, whereas Mann–Whitney U and Kruskal–Wallis tests were used for variables that were not normally distributed. No formal adjustment for multiple testing was applied to the correlation, multivariable regression, or mediation analyses; therefore, the reported *p*-values were unadjusted for multiple comparisons. Associations between continuous variables were assessed using Spearman’s correlation coefficients, and correlation heatmaps were generated in R for data visualization. Multivariable linear regression analyses were performed to evaluate associations between BPA exposure variables and oxidative stress biomarkers, inflammatory biomarkers, and fetal and neonatal outcomes. Robust regression analyses were additionally performed using the MASS package in R for outcome variables that did not meet normality assumptions or were influenced by outliers. Regression models were adjusted for potential confounding variables, including maternal age, gestational age, pre-pregnancy BMI, parity, infant sex, energy intake, urinary creatinine, DII, and DTAC, as appropriate for each outcome and exposure model. Mediation analyses evaluating direct and indirect effects were conducted using SmartPLS software (version 4.0). Statistical significance was assessed using *p*-values, with *p* < 0.05 considered statistically significant.

### 2.9. Use of Artificial Intelligence Tools

OpenAI’s ChatGPT (GPT-5, accessed via ChatGPT Plus) was used for English-language editing and refinement of the manuscript and to assist with the design and layout of the graphical abstract. All AI-assisted outputs were reviewed and edited by the authors, who take full responsibility for the content of the manuscript and the graphical abstract.

## 3. Results

Table 1 presents the baseline sociodemographic, clinical, fetal, and neonatal characteristics of the study population. The participants had a mean age of 29.2 ± 4.3 years and a mean pre-pregnancy BMI of 24.10 ± 3.84 kg/m^2^. Most participants had attained university-level education (67.5%), were not employed (59.3%), and were nulliparous (58.1%). Fetal and neonatal anthropometric characteristics are also summarized in Table 1.

Daily energy and nutrient intakes of participants, as assessed by the FFQ, are summarized in Table 2. The mean daily energy intake was 2093.6 ± 488.9 kcal. The mean percentages of energy derived from fat, carbohydrate, and protein were 42.7 ± 6.7%, 42.2 ± 6.9%, and 15.1 ± 2.1%, respectively. The mean dietary fiber intake was 31.9 ± 11.1 g/day. The mean dietary antioxidant capacity was 5.66 ± 1.62 mmol/1000 kcal, and the median Dietary Inflammatory Index (DII) score was −2.33 (−3.09 to −1.08).

Daily food group intakes assessed by 3-day dietary records and the FFQ are presented in Supplementary Table S1 and provide contextual information on the dietary data used to estimate BPA exposure. Estimated dietary BPA exposure by food groups based on 3-day dietary records and FFQ data is presented in Table 3. Total dietary BPA intake was 0.0203 (0.0161–0.0273) µg/kg/day based on 3-day dietary records and 0.0246 (0.0202–0.0323) µg/kg/day based on FFQ data. Estimated BPA exposure from meat and meat products, and vegetables and fruits, showed higher values compared with other food groups in both dietary assessment methods. Descriptive statistics of maternal urinary BPA levels, oxidative stress, and inflammatory biomarkers are provided in Supplementary Table S2. Associations between BPA-related dietary behaviors and urinary BPA concentrations are presented in Supplementary Table S3. No significant differences in urinary BPA concentrations were observed across eating-out frequency, fast-food consumption, packaged-food consumption, or canned-food consumption behaviors (all *p* > 0.05). Correlations between estimated dietary BPA exposure and urinary BPA concentrations are provided in Supplementary Table S4.

**Figure 2:**
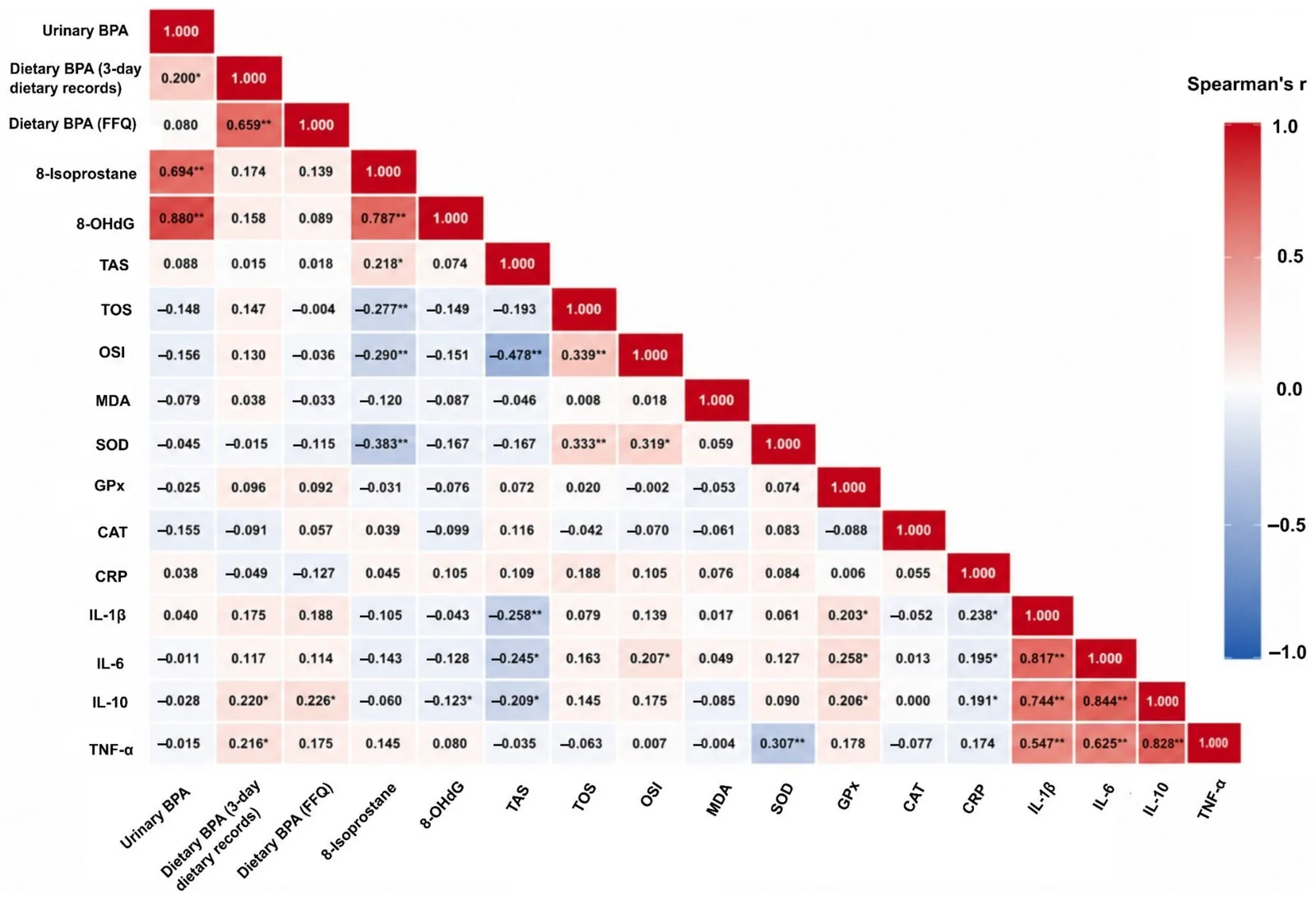
Correlation matrix of maternal BPA exposure, oxidative stress, and inflammatory biomarkers. Cells display Spearman correlation coefficients (*r*), and the color gradient indicates the strength and direction of the correlations. Significance: * *p* < 0.05 and ** *p* < 0.001. Urinary BPA concentrations and urinary biomarkers (8-isoprostane and 8-OHdG) are presented as creatinine-adjusted values (µg/g creatinine). Abbreviations: BPA, bisphenol A; FFQ, Food Frequency Questionnaire; TAS, total antioxidant status; TOS, total oxidant status; OSI, oxidative stress index; MDA, malondialdehyde; SOD, superoxide dismutase; GPx, glutathione peroxidase; CAT, catalase; CRP, C-reactive protein; IL-1β, interleukin-1 beta; IL-6, interleukin-6; IL-10, interleukin-10; TNF-α, tumor necrosis factor-alpha; 8-OHdG, 8-hydroxy-2′-deoxyguanosine.

Correlations between urinary BPA levels (µg/g creatinine), estimated dietary BPA intake, and oxidative stress and inflammatory biomarkers are presented in Figure 2 and Supplementary Table S5. Urinary BPA levels showed strong positive correlations with urinary 8-isoprostane (*r* = 0.694, *p* < 0.001) and urinary 8-OHdG (*r* = 0.880, *p* < 0.001). No significant correlations were observed between urinary BPA levels and serum oxidative stress parameters, antioxidant enzymes, or inflammatory biomarkers (all *p* > 0.05). Estimated dietary BPA intake based on 3-day dietary records showed positive correlations with IL-10 (*r* = 0.221, *p* = 0.042) and TNF-α (*r* = 0.216, *p* = 0.046). Similarly, FFQ-based BPA intake was positively correlated with IL-10 levels (*r* = 0.226, *p* = 0.037).

Associations of maternal urinary BPA concentrations and dietary BPA exposure with oxidative stress and inflammatory biomarkers are presented in Table 4. In multivariable linear regression models adjusted for potential confounding variables, maternal urinary BPA concentrations were inversely associated with serum catalase levels in Model 1 (β = −0.21, *p* = 0.036). After additional adjustment for dietary inflammatory index and dietary total antioxidant capacity in Model 2, this association was no longer statistically significant (β = −0.19, *p* = 0.061). No significant associations were observed between urinary BPA concentrations and other oxidative stress or inflammatory biomarkers. Similarly, dietary BPA exposure was not significantly associated with oxidative stress or inflammatory biomarkers in either model. Although some significant associations were observed in the correlation analyses presented in Figure 2 and Supplementary Table S5, these associations were largely attenuated and generally lost statistical significance after adjustment for potential confounders in multivariable models.

**Figure 3:**
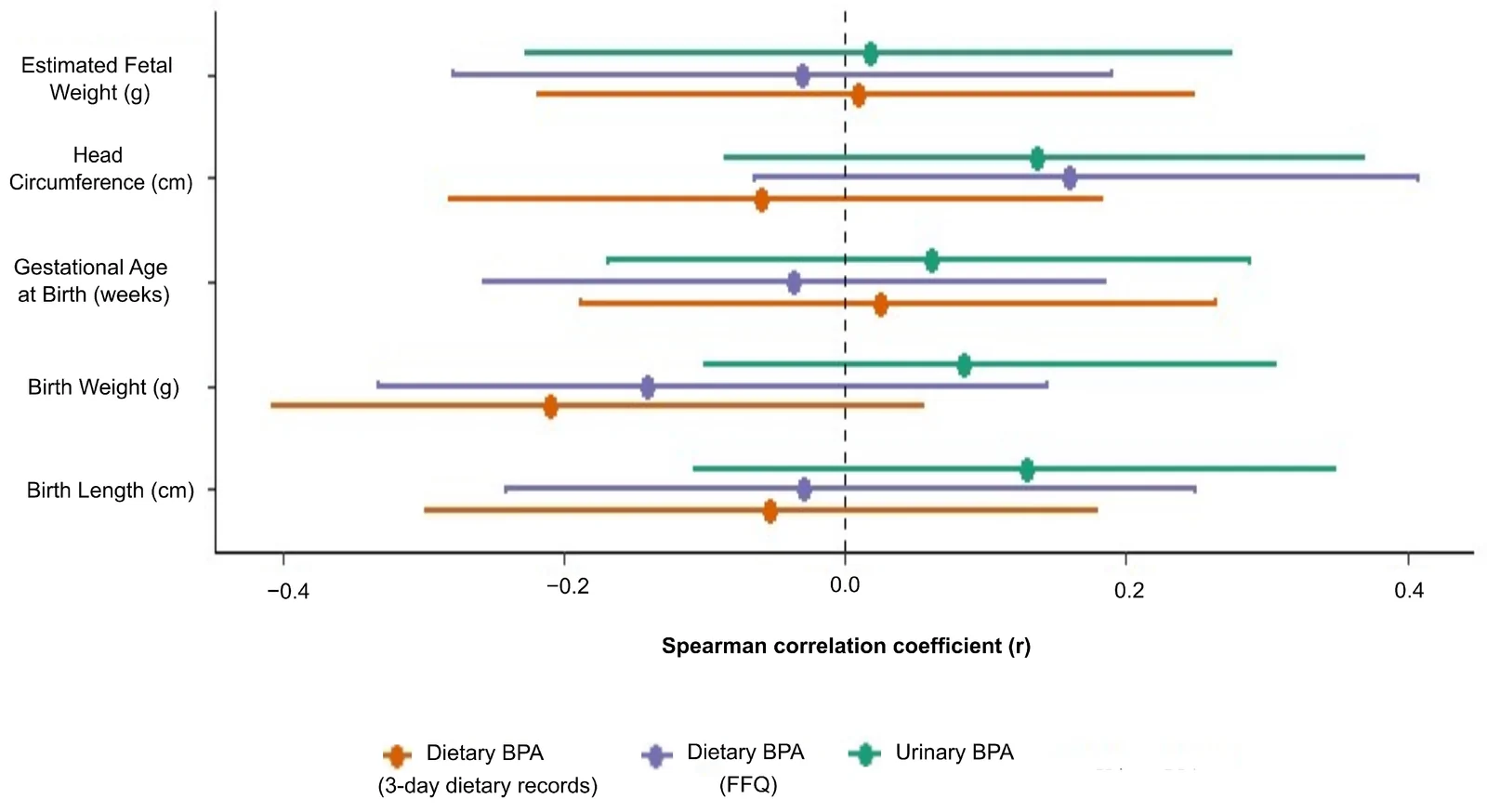
Forest plot of associations between maternal BPA exposure and fetal and neonatal outcomes. Points represent Spearman correlation coefficients (r), and horizontal lines indicate 95% confidence intervals. Confidence intervals crossing zero indicate non-significant associations. Urinary BPA concentrations are presented as creatinine-adjusted values (µg/g creatinine). Abbreviations: BPA, bisphenol A; FFQ, Food Frequency Questionnaire.

Correlations between maternal BPA exposure and fetal and neonatal outcomes are presented in Figure 3. Neither creatinine-adjusted urinary BPA concentrations nor dietary BPA intake estimated from 3-day dietary records or FFQ data was significantly correlated with EFW, birth weight, birth length, head circumference, or gestational age at birth (all *p* > 0.05). The correlation between dietary BPA intake estimated from 3-day dietary records and birth weight was inverse but did not reach statistical significance (*r* = −0.210, *p* = 0.054).

Multivariable linear regression analyses showed similar findings (Table 5). Maternal urinary BPA levels were not significantly associated with any of the evaluated fetal or neonatal outcomes after adjustment for potential confounders.

**Figure 4:**
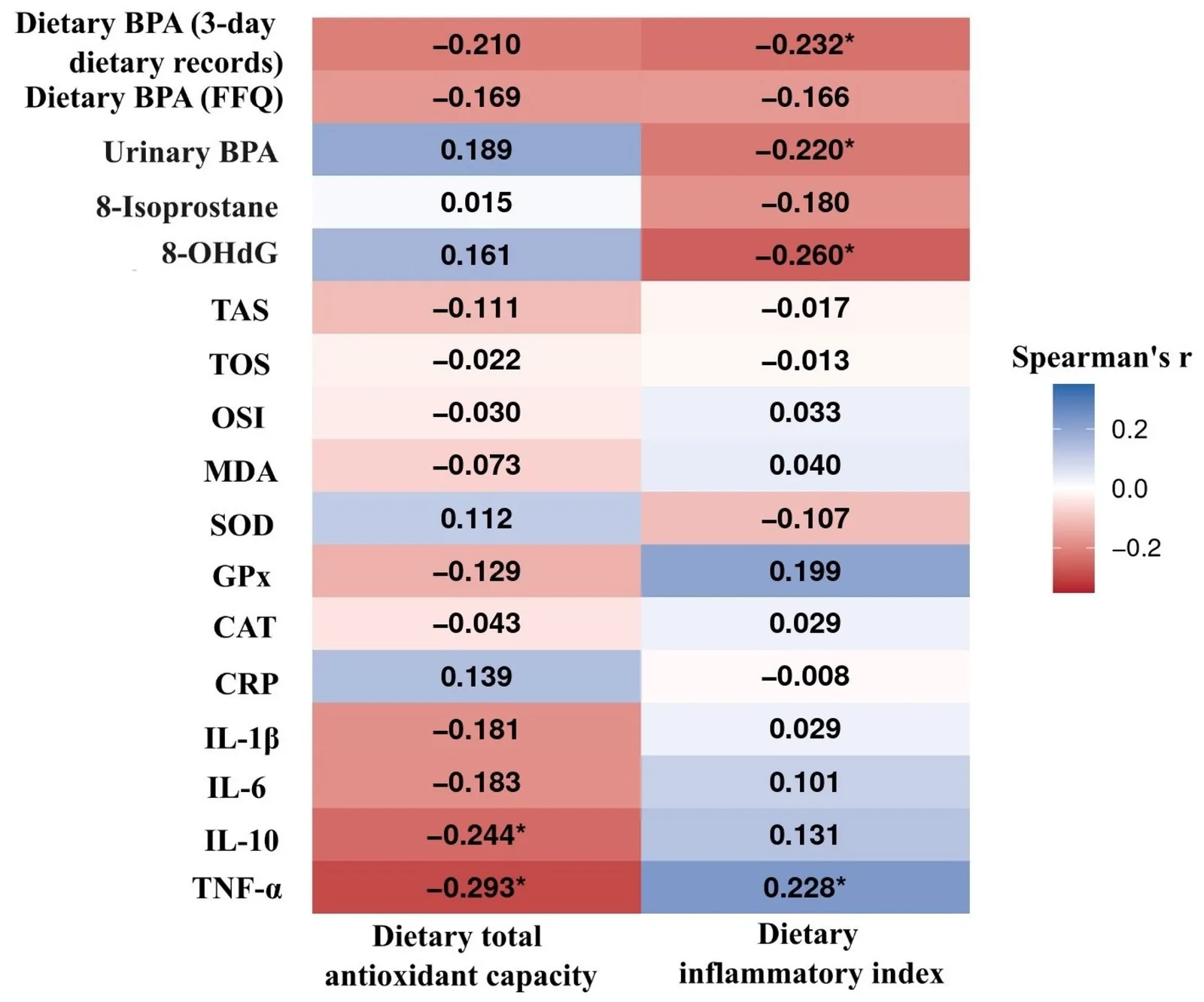
Correlations of dietary total antioxidant capacity and dietary inflammatory index with maternal BPA exposure and biomarkers of oxidative stress and inflammation. Cells display Spearman correlation coefficients (r), and the color gradient indicates the strength and direction of the correlations. Significance: * *p* < 0.05. Urinary BPA concentrations and urinary biomarkers (8-isoprostane and 8-OHdG) are presented as creatinine-adjusted values (µg/g creatinine). Abbreviations: BPA, bisphenol A; FFQ, Food Frequency Questionnaire; DTAC, dietary total antioxidant capacity; DII, dietary inflammatory index; TAS, total antioxidant status; TOS, total oxidant status; OSI, oxidative stress index; MDA, malondialdehyde; SOD, superoxide dismutase; GPx, glutathione pe-roxidase; CAT, catalase; CRP, C-reactive protein; IL-1β, interleukin-1 beta; IL-6, interleukin-6; IL-10, interleu-kin-10; TNF-α, tumor necrosis factor-alpha; 8-OHdG, 8-hydroxy-2′-deoxyguanosine.

The correlation patterns of DTAC and DII with BPA exposure, oxidative stress, and inflammatory biomarkers are illustrated in Figure 4, with the corresponding correlation coefficients and *p*-values provided in Supplementary Table S6. DTAC was inversely correlated with IL-10 (*r* = −0.244, *p* = 0.024) and TNF-α (*r* = −0.293, *p* = 0.006), whereas no significant correlations were observed with the remaining biomarkers. DII was negatively correlated with creatinine-adjusted urinary BPA levels (*r* = −0.232, *p* = 0.032), FFQ-based BPA intake (*r* = −0.220, *p* = 0.042), and creatinine-adjusted urinary 8-OHdG levels (*r* = −0.260, *p* = 0.016), while a positive correlation was observed with TNF-α (*r* = 0.228, *p* = 0.035).

Mediation analyses examining the potential indirect effects of oxidative stress and inflammatory biomarkers on the associations between BPA exposure and fetal/neonatal outcomes are presented in Supplementary Tables S7 and S8. No significant indirect effects were observed for any of the evaluated mediators.

Additional multivariable analyses showed that dietary SFA, MUFA, and PUFA intakes were not significantly associated with urinary 8-isoprostane or 8-OHdG concentrations (all *p* > 0.05; Supplementary Table S9).

Estimated dietary BPA exposure and HQ values are presented in Table 6. According to the current reference values established in 2023, HQ values exceeded 1 for both 3-day dietary record–based and FFQ-based BPA exposure estimates. In contrast, HQ values calculated using the earlier 2015 reference values remained below 1. Similar trends were observed for median and high-percentile (P95) exposure estimates.

## 4. Discussion

This study evaluated maternal urinary and dietary BPA exposure in relation to urinary biomarkers of oxidative damage, serum biomarkers of oxidative stress and inflammation, and fetal and neonatal outcomes during pregnancy. The principal finding of this study was that creatinine-adjusted urinary BPA concentrations were strongly and positively correlated with urinary 8-isoprostane and 8-OHdG in correlation analyses; however, these associations were no longer statistically significant in multivariable models adjusted for potential confounders. An inverse association between urinary BPA and serum catalase was observed after adjustment for potential confounders; however, this association was attenuated and no longer statistically significant after further adjustment for DII and DTAC. No other adjusted associations of urinary or dietary BPA exposure with oxidative stress or inflammatory biomarkers were statistically significant. Similarly, no associations were observed with fetal or neonatal anthropometric outcomes. Mediation analyses further indicated that oxidative stress and inflammatory biomarkers did not account for the relationship between BPA exposure and fetal outcomes. Notably, diet-related indices, including dietary total antioxidant capacity and the Dietary Inflammatory Index, were associated with selected inflammatory biomarkers, suggesting that dietary characteristics may contribute to variability in inflammatory status within this population. The strong correlations observed between urinary BPA levels and urinary biomarkers of oxidative damage warrant further investigation of underlying biological mechanisms. Urinary 8-OHdG and 8-isoprostane are widely recognized as indicators of oxidative damage to DNA and lipids, respectively, resulting from ROS generation [60,61]. As these oxidized compounds are excreted in urine and reflect the overall burden of oxidative damage, urinary measurements are considered sensitive indicators of exposure-related oxidative stress [62]. Experimental research using primary human endometrial stromal cells has shown that BPA exposure increases ROS generation and activates MAPK- and NF-κB-related inflammatory signaling [63]. Consistent with the present findings, studies conducted during pregnancy have reported positive associations between urinary BPA and the urinary oxidative damage biomarkers 8-OHdG and 8-isoprostane, whereas associations with circulating inflammatory biomarkers were largely null in Watkins et al. (2015) [64] and were primarily limited to IL-6 in Ferguson et al. (2016) [18]. Circulating biomarkers of oxidative stress and inflammation are also subject to homeostatic regulation and dynamic physiological fluctuations [65,66]. Longitudinal data further indicate that urinary 8-isoprostane decreases slightly, whereas urinary 8-OHdG increases as pregnancy progresses [67], and that maternal serum cytokines follow heterogeneous gestational trajectories [68]. Thus, differences in the biological processes reflected by these biomarkers, the sample types in which they are measured, and their changes across pregnancy may partly account for the more evident associations observed for urinary oxidative damage markers than for serum biomarkers.

Although significant associations between urinary BPA levels and urinary markers of oxidative damage were observed in the correlation analyses, these relationships were largely attenuated after adjustment for potential confounders in the multivariable models. This attenuation may reflect interindividual differences in biological responsiveness and toxicological susceptibility, as well as the influence of nutritional factors on oxidative stress and inflammation [69,70,71]. In addition, both oxidative stress and inflammatory processes are influenced by multiple biological and environmental factors, potentially contributing to variability in biomarker responses and complicating the identification of consistent associations [65,72]. In Model 1, urinary BPA was inversely associated with serum catalase; however, this association was no longer statistically significant after DII and DTAC were added to Model 2. DII and DTAC reflect the overall inflammatory potential and antioxidant capacity of the diet, respectively, and are composite indices representing multiple dietary components. Because these indices were added to the model simultaneously, the difference between Models 1 and 2 cannot be attributed to a specific nutrient or interpreted as evidence that diet neutralized BPA-related oxidative effects. Experimental studies have reported alterations in catalase activity following BPA exposure [73,74], suggesting that BPA exposure may affect antioxidant defense mechanisms. However, these studies do not establish that the difference between Models 1 and 2 was attributable to dietary factors.

In the present study, estimated dietary BPA exposure showed positive correlations with IL-10 and TNF-α in the correlation analyses. However, the multivariable models presented in Table 4 showed no statistically significant associations between dietary BPA exposure and the evaluated biomarkers. These correlation findings should therefore be interpreted in conjunction with the adjusted analyses. Previous human studies evaluating BPA exposure have also reported associations with inflammatory and immune-related biomarkers, suggesting that bisphenol exposure may influence inflammatory and immune systems under certain physiological conditions [18,75,76]. Experimental animal studies have also reported alterations in inflammatory cytokines, oxidative stress markers, and antioxidant defense systems in relation to dietary BPA exposure [77,78]. In the present study, meat and meat products, as well as vegetables and fruits, were the major contributors to estimated dietary BPA exposure across both dietary assessment methods. Similar findings have been reported in previous dietary exposure assessments, suggesting that cumulative intake from commonly consumed food groups may substantially contribute to overall BPA exposure, even when BPA concentrations in individual foods are relatively low [44,79]. Median creatinine-adjusted urinary BPA concentrations observed in the present cohort were within the range previously reported among pregnant women in biomonitoring studies, although substantial variability across populations has been described depending on dietary habits, geographical region, and exposure assessment methods [80,81].

In the present study, dietary BPA estimates were not significantly correlated with urinary BPA concentrations (Supplementary Table S4). The 3-day dietary records and FFQ provided complementary estimates of short-term and habitual dietary intake, respectively. However, in both dietary assessments, BPA exposure was estimated from self-reported intake and published BPA concentration values rather than by direct chemical analysis of the foods actually consumed. In addition, a single BPA measurement in a spot urine sample reflects recent exposure from both dietary and non-dietary sources [82,83] and is subject to considerable within-person variability during pregnancy [84,85]. Non-dietary BPA sources were not assessed in the present study. Therefore, the absence of a correlation should not be interpreted as evidence that dietary intake does not contribute to BPA exposure; rather, it may reflect the indirect nature of dietary exposure estimation, within-person variability in urinary BPA concentrations, and unmeasured non-dietary exposure. Additional analyses evaluating BPA-related dietary behaviors, including consumption of ready-to-eat packaged foods, use of canned foods, and frequency of eating out, did not demonstrate significant associations with creatinine-adjusted urinary BPA concentrations (Supplementary Table S3). Previous studies examining BPA-related dietary behaviors have reported inconsistent findings, and differences in study populations, including variations in age and sex composition, as well as methodological approaches used to assess BPA exposure, may partly explain these discrepancies [86,87,88].

Maternal urinary BPA levels and estimated dietary BPA intake were not significantly associated with fetal or neonatal anthropometric outcomes. Previous studies investigating BPA exposure and fetal growth outcomes have reported inconsistent findings, with some studies demonstrating associations with reduced birth weight, fetal growth restriction, or altered gestational duration [15,89,90], while others reported no significant associations [91,92,93,94]. These inconsistencies may partly reflect differences in the timing, frequency, and biological matrices used for BPA exposure assessment during pregnancy, as most previous studies evaluating fetal or neonatal outcomes have focused on urinary or serum BPA biomarkers rather than estimated dietary BPA exposure [95,96].

To further explore potential mechanisms underlying the relationship between BPA exposure and fetal and neonatal outcomes, mediation analyses were conducted for both maternal urinary BPA levels and estimated dietary BPA intake using oxidative stress and inflammatory biomarkers. However, no significant indirect effects were identified through the evaluated biomarkers. Although oxidative stress and inflammatory pathways have been proposed as biologically possible mechanisms underlying BPA-related developmental effects [18,97], the present findings suggest that the evaluated biomarkers did not substantially mediate the relationship between BPA exposure and fetal and neonatal outcomes in this cohort. The absence of significant indirect effects may also reflect the use of single urine and serum measurements obtained during late pregnancy, which may not fully capture temporal variability in BPA exposure across gestation, particularly given the short biological half-life of BPA [82,95,98]. Moreover, BPA may exert developmental effects through multiple interconnected biological pathways, some of which may not have been fully captured by the evaluated biomarkers [99,100].

Similar findings have also been reported in a previous study, in which oxidative stress biomarkers did not significantly mediate the association between maternal BPA exposure and neonatal outcomes [101]. Unlike previous studies, the present study also incorporated dietary inflammatory and antioxidant indices, enabling a more comprehensive evaluation of potential oxidative and inflammatory mechanisms associated with BPA exposure.

Estimated dietary BPA exposure and HQ analyses demonstrated that HQ values exceeded 1 when calculated according to the updated EFSA reference values established in 2023, whereas HQ values remained below 1 according to the earlier 2015 reference values. This difference primarily reflects the substantial reduction in the TDI for BPA introduced by EFSA in 2023, following increasing evidence regarding potential immunological and endocrine-related health effects of BPA exposure. In this context, the finding that HQ values exceeded 1 under the updated reference value is consistent with EFSA’s recent conclusion that dietary BPA exposure may exceed the newly established TDI in some population groups [12]. A recent national-level study conducted in Türkiye using data from the nationally representative Turkey Nutrition and Health Survey also reported that estimated dietary BPA exposure exceeded the updated EFSA tolerable daily intake thresholds across multiple population groups [102]. These findings highlight the importance of continued monitoring of dietary BPA exposure, particularly in vulnerable populations such as pregnant women.

Correlation analyses evaluating maternal dietary antioxidant and inflammatory profiles demonstrated that higher DTAC levels were inversely associated with TNF-α concentrations, whereas higher DII scores were positively correlated with TNF-α levels. Inverse correlations were also observed between DTAC and IL-1β/IL-6, although these associations did not reach statistical significance. These findings are generally consistent with the proposed role of dietary antioxidant capacity and inflammatory potential in modulating inflammatory and oxidative stress pathways [31,103]. However, some unexpected associations were also observed, including inverse correlations between DTAC and IL-10 levels and between DII scores and creatinine-adjusted urinary 8-OHdG concentrations. Similar heterogeneity has been reported in studies evaluating dietary indices of inflammatory or oxidative stress in relation to inflammatory and oxidative stress biomarkers, suggesting that these indices may not show consistent associations across different biomarkers and study populations [104]. In pregnancy, associations between dietary patterns and inflammatory markers may also vary due to differences in dietary assessment methods, study design, analysis methods, and the inflammatory biomarkers evaluated [105]. In addition, the predominantly anti-inflammatory dietary profile of the present cohort may have limited the variability of DII and DTAC values, making associations with certain biomarkers more difficult to detect.

DII scores were inversely correlated with estimated dietary BPA exposure and urinary BPA concentrations. The DII reflects the inflammatory potential of the diet based on multiple dietary components [31] but does not capture the packaging, processing, or storage conditions of the foods consumed. Although the BPA-related dietary behaviors evaluated in the present study were not significantly associated with creatinine-adjusted urinary BPA concentrations (Supplementary Table S3), the reported foods could not be individually linked to their actual packaging and storage conditions. BPA migration from food contact materials can vary according to food and packaging type, temperature, contact duration, processing, and storage conditions. In particular, high temperatures and sterilization processes, as well as certain prolonged-storage conditions, may increase BPA migration [8,12,106]. Therefore, fresh foods and foods contributing to a lower dietary inflammatory potential may nevertheless be purchased, processed, or stored in food contact materials from which BPA migration can occur. However, this possibility could not be directly evaluated using the available data, and the observed inverse correlations cannot be attributed to a specific packaging-related behavior.

Participants with known chronic chemical exposure, smoking or second-hand smoke exposure, or occupations involving potential chemical exposure, such as cashier or textile work, were excluded from the study. Nevertheless, other potential non-dietary sources of BPA exposure, including contact with thermal paper receipts, household dust, clothing, cosmetics and personal-care products, and cleaning products, were not specifically quantified [8,12,107,108]. These unmeasured environmental and consumer-product-related sources may therefore have contributed to the observed urinary BPA concentrations.

Diet-related factors were also considered as potential confounders of the correlations between urinary BPA and urinary oxidative stress biomarkers. In this context, dietary fatty acid intake was examined as one possible factor contributing to variation in these biomarkers. This consideration was particularly relevant to 8-isoprostane because F_2_-isoprostanes are generated through free radical-induced peroxidation of arachidonic acid [109,110]. However, total PUFA intake represents a composite measure of several fatty acids rather than a specific estimate of arachidonic acid intake; therefore, the direction of the association with 8-isoprostane could not be inferred from total PUFA intake alone. In the fully adjusted models, which included DII and DTAC, the associations of urinary BPA with urinary 8-isoprostane and 8-OHdG were not retained. To examine the fatty acid-related explanation further, dietary SFA, MUFA, and PUFA intakes were evaluated in separate multivariable models. After adjustment for maternal age, gestational week, pre-pregnancy BMI, total energy intake, and urinary creatinine, none of these fatty acid classes was significantly associated with urinary 8-isoprostane or 8-OHdG (Supplementary Table S9). Thus, the present analyses did not support total dietary SFA, MUFA, or PUFA intake as an explanation for the strong correlations observed between urinary BPA and these urinary biomarkers. Conversely, the correlations alone cannot be interpreted as evidence of a direct effect of BPA on oxidative damage.

These analyses assessed dietary intake rather than circulating fatty acid status. In a validation study among pregnant women, questionnaire-based intake estimates showed moderate correlations with plasma concentrations for EPA and DHA, a weak correlation for α-linolenic acid, and no correlation for linoleic or arachidonic acid [111]. Because plasma fatty acid composition was not measured in the present study, the possible contribution of specific circulating fatty acid profiles could not be evaluated. Furthermore, the absence of corresponding BPA-related associations across the serum oxidative stress biomarker panel suggests that the findings did not form a consistent pattern across biological matrices. However, this should not be interpreted as evidence that the urinary findings were caused solely by dietary factors or that systemic oxidative stress and cellular damage were absent, because the biomarkers examined reflect different molecular targets and have distinct biological and measurement characteristics [66]. Urinary 8-isoprostane and 8-OHdG concentrations may also vary across pregnancy [67]. Taken together, these findings underscore the complexity of the relationships between maternal BPA exposure, dietary characteristics, oxidative stress, and inflammatory responses during pregnancy. Although the present study provides valuable insights into the interplay between maternal BPA exposure, dietary characteristics, oxidative stress, and inflammatory responses during pregnancy, its cross-sectional design precludes evaluation of temporal changes and causal relationships.

## 5. Strengths and Limitations

This study has several important strengths. Maternal BPA exposure was evaluated using urinary BPA measurements with consideration of urinary creatinine and estimated dietary BPA intake derived from 3-day dietary records and an FFQ, providing complementary biomarker-based and diet-based exposure estimates. Additional strengths include the comprehensive assessment of oxidative stress, antioxidant defense, and inflammatory biomarkers; inclusion of the DII and DTAC; evaluation of fetal and neonatal anthropometric outcomes; and application of mediation analyses.

Several limitations should also be considered. The descriptive cross-sectional design precludes conclusions regarding temporality or causality. Urinary BPA was assessed using a single first-morning urine sample. Although first-morning collection improved consistency and urinary creatinine was used to account for urine dilution, these measures could not capture day-to-day variability in BPA exposure or fully account for physiological variation in creatinine excretion during pregnancy. Given BPA’s short biological half-life, a single measurement primarily reflects recent exposure and may not represent usual exposure during late pregnancy. Moreover, urinary BPA, oxidative stress, and inflammatory biomarkers were measured only once during the third trimester, preventing assessment of variability across gestation and associations during potentially sensitive earlier exposure windows. Repeated urine measurements across pregnancy, ideally using multiple spot urine samples and, where feasible, 24-h urine collections, would provide a more comprehensive assessment of exposure. However, such repeated biomonitoring approaches require substantial financial resources.

Dietary BPA exposure was estimated from self-reported 3-day dietary records and FFQ data using published BPA concentration values rather than direct analysis of the foods consumed. Although these methods estimated short-term and habitual intake, both remained indirect and may not have captured variation in BPA concentrations among products; FFQ data may also have been affected by recall bias, whereas dietary records may have been subject to reporting or recording error. Foods were not individually linked to their packaging, processing, or storage conditions, and non-dietary BPA sources were not quantified. These limitations may have reduced the correspondence between estimated dietary BPA exposure and urinary BPA concentrations. Residual confounding by unmeasured environmental or lifestyle factors cannot be excluded. Iron, folic acid, and vitamin D supplement use was also highly prevalent, precluding an adequately sized non-supplemented comparison group and limiting the ability to assess the potential influence of supplement use on biomarker levels.

Plasma fatty acid composition was not measured; therefore, associations of circulating SFA, MUFA, and PUFA profiles with urinary oxidative stress biomarkers could not be evaluated. Because only BPA was measured, concurrent exposure to bisphenol S (BPS), bisphenol F (BPF), and other bisphenol analogues and their potential combined associations with maternal and fetal/neonatal outcomes could not be examined.

Although the final sample exceeded the minimum estimated by the a priori power analysis, its modest size may have limited estimate precision and the ability to detect small associations in multivariable regression and mediation analyses. Accordingly, statistically non-significant findings should not be regarded as definitive evidence of no association. Because multiple associations were evaluated without adjustment for multiple testing, the risk of type I error may have increased; isolated statistically significant findings, particularly those with *p*-values close to 0.05, should be interpreted in the context of the overall pattern of results and require confirmation in independent studies. Finally, recruitment of healthy pregnant women from a single tertiary hospital in Türkiye may limit generalizability. Larger multicenter studies involving more diverse populations are needed to estimate small effects more precisely and assess generalizability.

## 6. Future Research

Future longitudinal, multicenter studies involving larger and more diverse populations should prospectively assess bisphenol exposure, relevant oxidative stress and inflammatory biomarkers, and dietary characteristics throughout pregnancy. Given that the first and second trimesters encompass critical periods for fetal organogenesis and development, repeated measurements should be performed across all trimesters to characterize temporal exposure patterns and examine trimester-specific associations with fetal development and birth outcomes. Repeated urinary measurements across pregnancy, complemented where feasible by 24-h urine collections, should be combined with prospective assessment of dietary and non-dietary exposure sources and a comprehensive evaluation of overall diet quality, including relevant dietary indices. Future studies should also simultaneously measure BPA and its analogues, including BPS and BPF, and apply mixture-based analytical approaches to evaluate the potential combined effects of exposure to multiple bisphenols. To elucidate biological pathways beyond oxidative stress and inflammation, placental gene-regulatory and epigenetic alterations, including DNA methylation, should be investigated alongside long-term follow-up of offspring health. In addition, controlled intervention studies should determine whether practical BPA-reduction strategies, such as limiting the use of plastic food containers and other relevant food contact materials, reduce urinary BPA concentrations and improve oxidative stress biomarkers during pregnancy. Collectively, these approaches may clarify exposure pathways, temporal patterns, underlying biological mechanisms, and the potential implications of prenatal bisphenol exposure for maternal and child health.

## 7. Conclusions

In conclusion, creatinine-adjusted urinary BPA concentrations were strongly and positively correlated with urinary 8-isoprostane and 8-OHdG in the correlation analyses; however, these associations were not retained after adjustment for potential confounders. In Model 1, an inverse association was observed between urinary BPA and serum catalase, but this association was attenuated and no longer statistically significant in Model 2 after DII and DTAC were added. Taken together, these findings highlight the importance of considering potential confounders, dietary context, and biomarker type when evaluating associations of BPA exposure with oxidative stress and inflammatory biomarkers. Neither urinary nor dietary BPA exposure was significantly associated with fetal/neonatal outcomes, and the mediation analyses did not support a significant mediating role of the evaluated biomarkers in these relationships. DII and DTAC were correlated with selected inflammatory biomarkers, and dietary BPA hazard quotients calculated using the updated 2023 EFSA tolerable daily intake exceeded 1, indicating that estimated dietary BPA exposure warrants further evaluation and monitoring during pregnancy. The integrated evaluation of urinary and dietary BPA exposure, diet-related indices, and maternal biomarkers offers a comprehensive assessment of the complex interrelationships among these factors during late pregnancy and identifies priorities for future longitudinal studies incorporating repeated measurements of exposure and biomarkers.

## Figures and Tables

**Table 1:** Baseline characteristics of the study participants (n = 86).

Variables		Value
Age (years), mean ± SD		29.2 ± 4.3
Age group (years), n (%)	19–28	40 (46.5)
	29–40	46 (53.5)
Education level, n (%)	High school or below	28 (32.6)
	University or above	58 (67.4)
Employment status, n (%)	Working	35 (40.7)
	Not working	51 (59.3)
Pre-pregnancy weight (kg), mean ± SD		63.33 ± 10.33
Height (cm), mean ± SD		162.0 ± 5.0
Current weight (kg), mean ± SD		74.00 ± 9.75
Pre-pregnancy BMI (kg/m^2^), mean ± SD		24.10 ± 3.84
Pre-pregnancy BMI category, n (%)	<18.5 (Underweight)	4 (4.7)
	18.5–24.9 (Normal weight)	53 (61.6)
	25.0–29.9 (Overweight)	20 (23.3)
	≥30 (Obese)	9 (10.5)
Gestational age at enrollment (weeks), mean ± SD		32.03 ± 3.00
Parity, n (%)	Nulliparous	50 (58.1)
	Multiparous	36 (41.9)
History of miscarriage/stillbirth, n (%)	Yes	13 (15.1)
	No	73 (84.9)
Estimated fetal weight (g), mean ± SD		2024.86 ± 698.9
Birth weight (g), mean ± SD		3214.82 ± 346
Birth length (cm), median (Q1–Q3)		50 (49–50.5)
Head circumference (cm), mean ± SD		34.63 ± 1.85
Gestational age at birth (weeks), mean ± SD		38.7 ± 1.09

Abbreviations: BMI, body mass index. Data are presented as mean ± standard deviation (SD), median (Q1–Q3), or n (%), as appropriate.

**Table 2:** Daily energy and nutrient intake of participants based on FFQ.

Energy-Nutrients	Value
Energy (kcal), mean ± SD	2093.59 ± 488.94
Protein (g), mean ± SD	76.72 ± 21.09
Plant Protein (g), mean ± SD	39.82 ± 11.98
Animal Protein (g), mean ± SD	36.9 ± 13.87
Protein (%), mean ± SD	15.08 ± 2.14
Fat (g), mean ± SD	98.65 ± 23.03
Fat (%), mean ± SD	42.69 ± 6.68
Carbohydrate (g), mean ± SD	217.53 ± 71.15
Carbohydrate (%), mean ± SD	42.24 ± 6.86
Dietary Fiber (g), mean ± SD	31.95 ± 11.06
Saturated Fat (g), mean ± SD	30.07 ± 8.21
MUFA (g), mean ± SD	35.99 ± 10.73
PUFA (g), mean ± SD	25.19 ± 8.85
Cholesterol (mg), mean ± SD	285.99 ± 105.99
Omega-3 Fatty Acids (g), median (Q1–Q3)	2.41 (1.57–3.55)
Omega-6 Fatty Acids (g), mean ± SD	22.26 ± 7.74
Omega-6/Omega-3 Ratio, median (Q1–Q3)	8.5 (6.33–11.5)
DTAC (mmol/1000 kcal), mean ± SD	5.66 ± 1.62
Vitamin A (μg), median (Q1–Q3)	1611.04 (1277.34–2047.64)
Retinol (μg), median (Q1–Q3)	353.39 (285.22–436.17)
β-Carotene (µg), median (Q1–Q3)	6.81 (5.16–9.13)
Vitamin D (µg), median (Q1–Q3)	2.35 (1.63–4.08)
Vitamin E (mg), mean ± SD	23.84 ± 7.67
Vitamin K (μg), median (Q1–Q3)	184.28 (109.7–259.06)
Thiamine (mg), mean ± SD	1.35 ± 0.38
Riboflavin (mg), mean ± SD	1.87 ± 0.49
Niacin (mg), median (Q1–Q3)	13.97 (11.03–17.91)
Vitamin B_6_ (mg), median (Q1–Q3)	1.91 (1.56–2.21)
Total Folate (μg), mean ± SD	486.19 ± 151.57
Vitamin B_12_ (μg), mean ± SD	3.99 ± 1.79
Vitamin C (mg), median (Q1–Q3)	188.33 (136.5–246.14)
Potassium (mg), mean ± SD	3953.11 ± 1152.6
Calcium (mg), mean ± SD	1015.84 ± 320.58
Magnesium (mg), mean ± SD	418.07 ± 121.08
Phosphorus (mg), mean ± SD	1399.65 ± 381.2
Iron (mg), mean ± SD	14.72 ± 4.61
Zinc (mg), mean ± SD	12.01 ± 3.53
Copper (mg), mean ± SD	2.36 ± 0.71
Manganese (mg), median (Q1–Q3)	8.91 (6.79–16.59)
Soluble Fiber (g), mean ± SD	9.45 ± 3.39
Insoluble Fiber (g), mean ± SD	20.81 ± 7.36
Eugenol (mg), median (Q1–Q3)	0 (0–0)
Flavonols (mg), median (Q1–Q3)	35.8 (27.2–46.89)
Isoflavones (mg), median (Q1–Q3)	0.09 (0.07–0.18)
Flavones (mg), median (Q1–Q3)	4.91 (2.48–13.03)
Flavanones (mg), median (Q1–Q3)	20.28 (10.88–44.68)
Flavan-3-ols (mg), median (Q1–Q3)	270.66 (124.85–368.25)
Anthocyanidins (mg), median (Q1–Q3)	8.46 (5.23–17.23)
Caffeine (mg), median (Q1–Q3)	59 (23.28–80.85)
Selenium (μg), mean ± SD	13.18 ± 6.56
DII, median (Q1–Q3)	−2.33 (−3.09–−1.08)

Abbreviations: DTAC, dietary total antioxidant capacity; DII, Dietary Inflammatory Index; MUFA, monounsaturated fatty acids; PUFA, polyunsaturated fatty acids; SD, standard deviation.

**Table 3:** Estimated dietary BPA exposure by food groups based on 3-day dietary records and FFQ (μg/kg/day).

Food Groups	3-Day Dietary Records Median (Q_1_–Q_3_)	FFQ Median (Q_1_–Q_3_)
Milk and dairy products	0.0009 (0.0006–0.0013)	0.0012 (0.0008–0.0015)
Meat and meat products	0.0069 (0.0045–0.0109)	0.0063 (0.0038–0.0103)
Vegetables and fruits	0.0055 (0.004–0.008)	0.0088 (0.0067–0.0108)
Bread and cereals	0.0027 (0.002–0.0038)	0.0021 (0.0014–0.0029)
Fats and oils	0.0003 (0.0002–0.0004)	0.0002 (0.0002–0.0003)
Sweets	0.0001 (0–0.0003)	0.0002 (0.0001–0.0003)
Non-alcoholic beverages	0.0005 (0.0002–0.0009)	0.0011 (0.0006–0.0015)
Other (sauces, canned foods, tomato paste)	0.0002 (0–0.0029)	0.0013 (0.0002–0.008)
Total estimated dietary BPA intake	0.0203 (0.0161–0.0273)	0.0246 (0.0202–0.0323)

Abbreviations: BPA, bisphenol A; FFQ, Food Frequency Questionnaire.

**Table 4:** Adjusted associations between maternal BPA exposure and oxidative stress and inflammatory biomarkers.

	Urinary BPA				Dietary BPA Exposure			
	Model 1		Model 2		Model 1		Model 2	
Outcome Variables	β (95% CI)	*p*	β (95% CI)	*p*	β (95% CI)	*p*	β (95% CI)	*p*
**Urinary Biomarkers**								
8-Isoprostane	−0.04 (−0.27, 0.20)	0.722	−0.03 (−0.26, 0.23)	0.842	0.24 (−0.05, 0.52)	0.096	0.28 (−0.03, 0.55)	0.064
8-OHdG	0.04 (−0.13, 0.25)	0.672	0.02 (−0.16, 0.23)	0.852	−0.17 (−0.44, 0.12)	0.246	−0.17 (−0.42, 0.10)	0.220
**Serum Biomarkers**								
TAS	0.09 (−0.14, 0.31)	0.449	0.11 (−0.10, 0.33)	0.320	0.04 (−0.29, 0.38)	0.835	0.02 (−0.29, 0.38)	0.892
TOS	−0.14 (−0.34, 0.08)	0.209	−0.15 (−0.37, 0.10)	0.201	0.10 (−0.20, 0.40)	0.520	0.13 (−0.16, 0.44)	0.410
OSI	−0.14 (−0.35, 0.08)	0.198	−0.16 (−0.37, 0.10)	0.186	0.08 (−0.21, 0.38)	0.584	0.12 (−0.18, 0.42)	0.466
MDA	−0.19 (−0.42, 0.13)	0.169	−0.19 (−0.42, 0.15)	0.187	−0.07 (−0.30, 0.17)	0.551	−0.08 (−0.35, 0.20)	0.557
SOD	0.03 (−0.20, 0.26)	0.777	0.03 (−0.21, 0.26)	0.822	−0.15 (−0.39, 0.07)	0.189	−0.10 (−0.35, 0.16)	0.452
GPx	−0.05 (−0.23, 0.14)	0.596	−0.04 (−0.21, 0.13)	0.676	0.15 (−0.09, 0.41)	0.250	0.05 (−0.18, 0.33)	0.705
CAT	−0.21 (−0.40, −0.02)	**0.036**	−0.19 (−0.38, 0.01)	0.061	0.13 (−0.11, 0.36)	0.258	0.17 (−0.09, 0.41)	0.167
CRP	−0.05 (−0.26, 0.15)	0.618	−0.05 (−0.28, 0.16)	0.627	−0.12 (−0.32, 0.11)	0.281	−0.12 (−0.34, 0.13)	0.310
IL−1β	−0.03 (−0.26, 0.19)	0.770	0.00 (−0.23, 0.22)	0.999	0.09 (−0.18, 0.39)	0.536	0.16 (−0.09, 0.43)	0.239
IL−6	0.00 (−0.22, 0.21)	0.998	0.03 (−0.18, 0.25)	0.771	0.02 (−0.24, 0.30)	0.888	0.08 (−0.15, 0.34)	0.499
IL−10	−0.01 (−0.23, 0.20)	0.943	0.03 (−0.18, 0.24)	0.757	0.13 (−0.17, 0.46)	0.404	0.19 (−0.08, 0.47)	0.178
TNF-α	−0.06 (−0.28, 0.18)	0.613	−0.02 (−0.24, 0.21)	0.848	0.19 (−0.14, 0.54)	0.276	0.20 (−0.09, 0.51)	0.184

Abbreviations: BPA, bisphenol A; 8-OHdG, 8-hydroxy-2′-deoxyguanosine; CI, confidence interval; TAS, total antioxidant status; TOS, total oxidant status; OSI, oxidative stress index; MDA, malondialdehyde; SOD, superoxide dismutase; GPx, glutathione peroxidase; CAT, catalase; CRP, C-reactive protein; IL-1β, interleukin-1 beta; IL-6, interleukin-6; IL-10, interleukin-10; TNF-α, tumor necrosis factor-alpha; FFQ, food frequency questionnaire; BMI, body mass index; DII, Dietary Inflammatory Index; DTAC, dietary total antioxidant capacity. Separate multivariable linear regression models were constructed for maternal urinary BPA concentrations and dietary BPA exposure variables. Dietary BPA exposure variables were derived from FFQ-based food consumption data. Model 1 was adjusted for age, gestational week, pre-pregnancy BMI, and energy intake. Urinary creatinine was additionally adjusted for in models involving urinary biomarkers. Model 2 was additionally adjusted for DII and DTAC. Standardized β coefficients with 95% confidence intervals are presented. Bold values indicate statistical significance (*p* < 0.05).

**Table 5:** Multivariable regression models for associations between maternal urinary BPA levels and fetal/neonatal outcomes.

Outcome Variables	β (95% CI)	*p*-Value	R^2^	F	*p* (Model)
Estimated fetal weight (g) *	−0.683 (−1.772, 0.407)	0.216	0.842	57.097	<0.001
Birth weight (g) *	−0.575 (−1.727, 0.576)	0.323	0.212	2.968	0.008
Gestational age (weeks) *	0.000 (−0.004, 0.004)	0.925	0.140	2.114	0.061
Birth length (cm) **	0.001 (−0.003, 0.004)	0.780	0.386	6.922	<0.001
Head circumference (cm) **	−0.002 (−0.006, 0.002)	0.346	0.386	3.360	0.004

* Lineer Regresyon; ** Robust Regresyon. Models were adjusted for selected covariates including age, pre-pregnancy BMI, gestational age, parity, birth sex, and urinary creatinine, as appropriate for each model. Variables showing multicollinearity were excluded from the final models. Male sex was used as the reference category for birth sex. The *p*-value represents the significance of the urinary BPA coefficient, whereas model *p* indicates overall model significance.

**Table 6:** Hazard quotient values for estimated dietary BPA exposure based on EFSA 2015 and 2023 reference values.

	Mean ± SD	Median (min–max)	P95
**Dietary BPA HQ (3-day dietary records)**			
2015	0.006 ± 0.003	0.01 (0–0.02)	0.01
2023	113.65 ± 50.86	101.59 (32.52–301.71)	205.141
**Dietary BPA HQ (FFQ)**			
2015	0.007 ± 0.003	0.01 (0–0.02)	0.013
2023	138.89 ± 65.69	123.23 (25.83–434.37)	258.36

Abbreviations: BPA, bisphenol A; HQ, hazard quotient; FFQ, Food Frequency Questionnaire; EFSA, European Food Safety Authority; SD, standard deviation; P95, 95th percentile. The 2015 and 2023 HQ values were calculated based on EFSA tolerable daily intake reference values.

## Data Availability

The data presented in this study are available from the corresponding author upon reasonable request. The data are not publicly available to protect participant privacy and confidentiality.

## Supplementary Materials

The following supporting information can be downloaded at https://www.mdpi.com/article/10.3390/nu18172836/s1, Table S1. Daily food group intake assessed by 3-day dietary records and food frequency questionnaire (FFQ); Table S2. Descriptive statistics of maternal urinary BPA concentrations and oxidative stress and inflammatory biomarkers; Table S3. Associations between BPA-related dietary behaviors and creatinine-adjusted urinary BPA concentrations; Table S4. Correlations between estimated dietary BPA exposure and urinary BPA concentrations; Table S5. Correlations between maternal BPA exposure, oxidative stress, and inflammatory biomarkers; Table S6. Correlations of dietary total antioxidant capacity and dietary inflammatory index with BPA exposure, oxidative stress, and inflammatory biomarkers; Table S7. Indirect effects of oxidative stress and inflammatory biomarkers on the associations between maternal urinary BPA levels and fetal and neonatal outcomes; Table S8. Indirect effects of oxidative stress and inflammatory biomarkers on the associations between maternal dietary BPA exposure and fetal and neonatal outcomes; Table S9. Adjusted associations between dietary fatty acid intake and urinary oxidative stress biomarkers. File S1: STROBE Statement—Checklist of items that should be included in reports of cross-sectional studies.

## Abbreviations

The following abbreviations are used in this manuscript:

8-OHdG	8-Hydroxy-2′-deoxyguanosine
BPA	Bisphenol A
BPF	Bisphenol F
BPS	Bisphenol S
CAT	Catalase
CRP	C-reactive protein
DII	Dietary inflammatory index
DTAC	Dietary total antioxidant capacity
EDCs	Endocrine-disrupting chemicals
EDI	Estimated daily intake
EFW	Estimated fetal weight
EFSA	European Food Safety Authority
FFQ	Food Frequency Questionnaire
GPx	Glutathione peroxidase
HQ	Hazard quotient
IL-1β	Interleukin-1 beta
IL-6	Interleukin-6
IL-10	Interleukin-10
MDA	Malondialdehyde
OSI	Oxidative stress index
ROS	Reactive oxygen species
SOD	Superoxide dismutase
TAS	Total antioxidant status
TNF-α	Tumor necrosis factor-alpha
TOS	Total oxidant status
SD	Standard deviation
SPSS	Statistical Package for the Social Sciences

## References

[1] Zoeller R.T., Brown T.R., Doan L.L., Gore A.C., Skakkebaek N.E., Soto A.M., Woodruff T.J., Vom Saal F.S. (2012). Endocrine-disrupting chemicals and public health protection: A statement of principles from The Endocrine Society. Endocrinology.

[2] Callaghan M.A., Alatorre-Hinojosa S., Connors L.T., Singh R.D., Thompson J.A. (2020). Plasticizers and Cardiovascular Health: Role of Adipose Tissue Dysfunction. Front. Pharmacol..

[3] Rochester J.R. (2013). Bisphenol A and human health: A review of the literature. Reprod. Toxicol..

[4] Mustieles V., D’Cruz S.C., Couderq S., Rodriguez-Carrillo A., Fini J.B., Hofer T., Steffensen I.L., Dirven H., Barouki R., Olea N. (2020). Bisphenol A and its analogues: A comprehensive review to identify and prioritize effect biomarkers for human biomonitoring. Environ. Int..

[5] Buoso E., Masi M., Limosani R.V., Oliviero C., Saeed S., Iulini M., Passoni F.C., Racchi M., Corsini E. (2025). Endocrine Disrupting Toxicity of Bisphenol A and Its Analogs: Implications in the Neuro-Immune Milieu. J. Xenobiot..

[6] Abraham A., Chakraborty P. (2020). A review on sources and health impacts of bisphenol A. Rev. Environ. Health.

[7] Liao C.Y., Kannan K. (2013). Concentrations and Profiles of Bisphenol A and Other Bisphenol Analogues in Foodstuffs from the United States and Their Implications for Human Exposure. J. Agric. Food Chem..

[8] Geens T., Aerts D., Berthot C., Bourguignon J.P., Goeyens L., Lecomte P., Maghuin-Rogister G., Pironnet A.M., Pussemier L., Scippo M.L. (2012). A review of dietary and non-dietary exposure to bisphenol-A. Food Chem. Toxicol..

[9] Bolognesi C., Castle L., Cravedi J.P., Engel K.H., Fowler P., Franz R., Grob K., Gürtler R., Husoy T., EFSA Panel on Food Contact Materials, Enzymes, Flavourings and Processing Aids (CEF) (2015). Scientific Opinion on the risks to public health related to the presence of bisphenol A (BPA) in foodstuffs: Part I—Exposure assessment. EFSA J..

[10] Casas M., Valvi D., Luque N., Ballesteros-Gomez A., Carsin A.E., Fernandez M.F., Koch H.M., Mendez M.A., Sunyer J., Rubio S. (2013). Dietary and sociodemographic determinants of bisphenol A urine concentrations in pregnant women and children. Environ. Int..

[11] Liu J., Wattar N., Field C.J., Dinu I., Dewey D., Martin J.W., APrON Study Team (2018). Exposure and dietary sources of bisphenol A (BPA) and BPA-alternatives among mothers in the APrON cohort study. Environ. Int..

[12] Lambre C., Barat Baviera J.M., Bolognesi C., Chesson A., Cocconcelli P.S., Crebelli R., Gott D.M., Grob K., Lampi E., EFSA Panel on Food Contact Materials, Enzymes and Processing Aids (CEP) (2023). Re-evaluation of the risks to public health related to the presence of bisphenol A (BPA) in foodstuffs. EFSA J..

[13] Wassenaar P.N.H., Trasande L., Legler J. (2017). Systematic Review and Meta-Analysis of Early-Life Exposure to Bisphenol A and Obesity-Related Outcomes in Rodents. Environ. Health Perspect..

[14] Lee J., Choi K., Park J., Moon H.B., Choi G., Lee J.J., Suh E., Kim H.J., Eun S.H., Kim G.H. (2018). Bisphenol A distribution in serum, urine, placenta, breast milk, and umbilical cord serum in a birth panel of mother-neonate pairs. Sci. Total Environ..

[15] Lee Y.M., Hong Y.C., Ha M., Kim Y., Park H., Kim H.S., Ha E.H. (2018). Prenatal Bisphenol-A exposure affects fetal length growth by maternal glutathione transferase polymorphisms, and neonatal exposure affects child volume growth by sex: From multiregional prospective birth cohort MOCEH study. Sci. Total Environ..

[16] Zhou B., Yang P., Deng Y.L., Zeng Q., Lu W.Q., Mei S.R. (2020). Prenatal exposure to bisphenol a and its analogues (bisphenol F and S) and ultrasound parameters of fetal growth. Chemosphere.

[17] Mustieles V., Williams P.L., Fernandez M.F., Mínguez-Alarcon L., Ford J.B., Calafat A.M., Hauser R., Messerlian C., Environment and Reproductive Health (EARTH) Study Team (2018). Maternal and paternal preconception exposure to bisphenols and size at birth. Hum. Reprod..

[18] Ferguson K.K., Cantonwine D.E., McElrath T.F., Mukherjee B., Meeker J.D. (2016). Repeated measures analysis of associations between urinary bisphenol-A concentrations and biomarkers of inflammation and oxidative stress in pregnancy. Reprod. Toxicol..

[19] Huang Y.F., Wang P.W., Huang L.W., Lai C.H., Yang W., Wu K.Y., Lu C.A., Chen H.C., Chen M.L. (2017). Prenatal Nonylphenol and Bisphenol A Exposures and Inflammation Are Determinants of Oxidative/Nitrative Stress: A Taiwanese Cohort Study. Environ. Sci. Technol..

[20] Yang Y.J., Hong Y.C., Oh S.Y., Park M.S., Kim H., Leem J.H., Ha E.H. (2009). Bisphenol A exposure is associated with oxidative stress and inflammation in postmenopausal women. Environ. Res..

[21] Gagné F. (2014). Oxidative Stress. Biochemical Ecotoxicology: Principles and Methods.

[22] Ferguson K.K., Kamai E.M., Cantonwine D.E., Mukherjee B., Meeker J.D., McElrath T.F. (2018). Associations between repeated ultrasound measures of fetal growth and biomarkers of maternal oxidative stress and inflammation in pregnancy. Am. J. Reprod. Immunol..

[23] Eick S.M., Ferguson K.K., Milne G.L., Rios-McConnell R., Velez-Vega C., Rosario Z., Alshawabkeh A., Cordero J.F., Meeker J.D. (2020). Repeated measures of urinary oxidative stress biomarkers and preterm birth in Puerto Rico. Free Radic. Biol. Med..

[24] Liang F., Huo X., Wang W., Li Y., Zhang J., Feng Y., Wang Y. (2020). Association of bisphenol A or bisphenol S exposure with oxidative stress and immune disturbance among unexplained recurrent spontaneous abortion women. Chemosphere.

[25] Clarke E.D., Stanford J., Ferguson J.J.A., Wood L.G., Collins C.E. (2023). Red Blood Cell Membrane Fatty Acid Composition, Dietary Fatty Acid Intake and Diet Quality as Predictors of Inflammation in a Group of Australian Adults. Nutrients.

[26] Santos S., Oliveira A., Lopes C. (2013). Systematic review of saturated fatty acids on inflammation and circulating levels of adipokines. Nutr. Res..

[27] Nani A., Murtaza B., Sayed Khan A., Khan N.A., Hichami A. (2021). Antioxidant and Anti-Inflammatory Potential of Polyphenols Contained in Mediterranean Diet in Obesity: Molecular Mechanisms. Molecules.

[28] Gil-Cardoso K., Gines I., Pinent M., Ardevol A., Terra X., Blay M. (2017). A cafeteria diet triggers intestinal inflammation and oxidative stress in obese rats. Br. J. Nutr..

[29] Sampey B.P., Vanhoose A.M., Winfield H.M., Freemerman A.J., Muehlbauer M.J., Fueger P.T., Newgard C.B., Makowski L. (2011). Cafeteria Diet Is a Robust Model of Human Metabolic Syndrome with Liver and Adipose Inflammation: Comparison to High-Fat Diet. Obesity.

[30] Cavicchia P.P., Steck S.E., Hurley T.G., Hussey J.R., Ma Y., Ockene I.S., Hebert J.R. (2009). A new dietary inflammatory index predicts interval changes in serum high-sensitivity C-reactive protein. J. Nutr..

[31] Shivappa N., Steck S.E., Hurley T.G., Hussey J.R., Hebert J.R. (2014). Designing and developing a literature-derived, population-based dietary inflammatory index. Public Health Nutr..

[32] Kyozuka H., Murata T., Fukuda T., Yamaguchi A., Yasuda S., Suzuki D., Kanno A., Sato A., Ogata Y., Hosoya M. (2022). Preconception dietary inflammatory index and hypertension disorders of pregnancy: The Japan environment and children’s study. Pregnancy Hypertens..

[33] Yin W.J., Yu L.J., Wu L., Zhang L., Li Q., Dai F.C., Tao R.X., Jiang X.M., Zhu P. (2022). Adequate 25(OH)D moderates the relationship between dietary inflammatory potential and cardiovascular health risk during the second trimester of pregnancy. Front. Nutr..

[34] Casas R., Castro-Barquero S., Crovetto F., Larroya M., Ruiz-Leon A.M., Segales L., Nakaki A., Youssef L., Benitez L., Casanovas-Garriga F. (2022). Maternal Dietary Inflammatory Index during Pregnancy Is Associated with Perinatal Outcomes: Results from the IMPACT BCN Trial. Nutrients.

[35] de Andrade Miranda D.E.G., Santos I.D.S., Silva C.A., Carvalho M.R., Shivappa N., Hebert J.R., Crivellenti L.C., Sartorelli D.S. (2022). Pro-inflammatory diet during pregnancy is associated with large-for-gestational-age infants. Nutr. Res..

[36] Souza M., Ferreira L.B., Dos Santos L.C. (2023). Dietary Inflammatory Index during pregnancy is associated with birth weight and child anthropometry up to 10 years old: A systematic review and meta-analysis. Nutr. Res..

[37] Sartorelli D.S., Carvalho M.R., da Silva Santos I., Crivellenti L.C., Souza J.P., Franco L.J. (2021). Dietary total antioxidant capacity during pregnancy and birth outcomes. Eur. J. Nutr..

[38] Shokri-Mashhadi N., Khoshhali M., Heidari-Beni M., Kelishadi R. (2022). Association between maternal plasma total antioxidant capacity and dietary antioxidants intake with birth size outcomes. J. Trop. Pediatr..

[39] Zujko M.E., Waskiewicz A., Witkowska A.M., Cicha-Mikolajczyk A., Zujko K., Drygas W. (2022). Dietary Total Antioxidant Capacity-A New Indicator of Healthy Diet Quality in Cardiovascular Diseases: A Polish Cross-Sectional Study. Nutrients.

[40] Pellegrini N., Vitaglione P., Granato D., Fogliano V. (2020). Twenty-five years of total antioxidant capacity measurement of foods and biological fluids: Merits and limitations. J. Sci. Food Agric..

[41] Prins J.R., Schoots M.H., Wessels J.I., Campmans-Kuijpers M.J.E., Navis G.J., van Goor H., Robertson S.A., van der Beek E.M., Sobrevia L., Gordijn S.J. (2022). The influence of the dietary exposome on oxidative stress in pregnancy complications. Mol. Asp. Med..

[42] Morales E., Garcia-Serna A.M., Larque E., Sanchez-Campillo M., Serrano-Munera A., Martinez-Gracia C., Santaella-Pascual M., Suarez-Martinez C., Vioque J., Noguera-Velasco J.A. (2022). Dietary Patterns in Pregnancy and Biomarkers of Oxidative Stress in Mothers and Offspring: The NELA Birth Cohort. Front. Nutr..

[43] Gassman N.R. (2017). Induction of oxidative stress by bisphenol A and its pleiotropic effects. Environ. Mol. Mutagen..

[44] Zhang H., Yang R., Shi W., Zhou X., Sun S. (2022). The association between bisphenol A exposure and oxidative damage in rats/mice: A systematic review and meta-analysis. Environ. Pollut..

[45] Baysal A., Aksoy M., Besler H.T., Bozkurt N., Keçecioğlu S., Mercanlıgil S.M., Kutluay Merdol T., Pekcan G., Yıldız E. (2018). Diyet El Kitabı.

[46] Rakıcıoğlu N., Acar Tek N., Ayaz A., Pekcan G. (2025). Food and Nutrition Photo Catalog: Dimensions and Quantities.

[47] Nutrition Information Systems (2021). Ebispro for Windows, Stuttgart, Germany.

[48] Haytowitz D.B., Wu X., Bhagwat S., U.S. Department of Agriculture, Agricultural Research Service (2018). Database for the Flavonoid Content of Selected Foods, Release 3.3. https://www.ars.usda.gov/ARSUserFiles/80400535/Data/Flav/Flav3.3.pdf.

[49] U.S. Department of Agriculture, Agricultural Research Service (2015). USDA Database for the Isoflavone Content of Selected Foods, Release 2.1. https://www.ars.usda.gov/ARSUserFiles/80400535/Data/isoflav/Isoflav_R2-1.pdf.

[50] Carlsen M.H., Halvorsen B.L., Holte K., Bøhn S.K., Dragland S., Sampson L., Willey C., Senoo H., Umezono Y., Sanada C. (2010). The total antioxidant content of more than 3100 foods, beverages, spices, herbs and supplements used worldwide. Nutr. J..

[51] Çiftçi S., Yalçın S.S., Samur G. (2021). Comparison of daily bisphenol A intake based on dietary and urinary levels in breastfeeding women. Reprod. Toxicol..

[52] Park J.H., Hwang M.S., Ko A., Jeong D.H., Lee J.M., Moon G., Lee K.S., Kho Y.H., Shin M.K., Lee H.S. (2016). Risk assessment based on urinary bisphenol A levels in the general Korean population. Environ. Res..

[53] Martinez M.A., Rovira J., Sharma R.P., Nadal M., Schuhmacher M., Kumar V. (2017). Prenatal exposure estimation of BPA and DEHP using integrated external and internal dosimetry: A case study. Environ. Res..

[54] Sungur Ş., Köroğlu M., Özkan A. (2014). Determination of bisphenol a migrating from canned food and beverages in markets. Food Chem..

[55] Lohman T.G., Roche A.F., Martorell R. (1988). Anthropometric Standardization Reference Manual.

[56] World Health Organization (2000). Obesity: Preventing and Managing the Global Epidemic: Report of a WHO Consultation.

[57] Yang M., Kim S.Y., Lee S.M., Chang S.S., Kawamoto T., Jang J.Y., Ahn Y.O. (2003). Biological monitoring of bisphenol A in a Korean population. Arch. Environ. Contam. Toxicol..

[58] Celik M., Iyigun I., Yalcin S.S., Cagan M., Yirun A., Cakir D.A., Tezel Yalcin H., Aliyev F., Celik H.T., Deren O. (2025). Bisphenol exposure in preterm neonates: A cohort study with measurements at admission and discharge in a neonatal intensive care unit in Ankara, Türkiye. BMC Pediatr..

[59] Jen J.F., Hsiao S.L., Liu K.H. (2002). Simultaneous determination of uric acid and creatinine in urine by an eco-friendly solvent-free high-performance liquid chromatographic method. Talanta.

[60] Graille M., Wild P., Sauvain J.J., Hemmendinger M., Guseva Canu I., Hopf N.B. (2020). Urinary 8-OHdG as a biomarker for oxidative stress: A systematic literature review and meta-analysis. Int. J. Mol. Sci..

[61] Graille M., Wild P., Sauvain J.J., Hemmendinger M., Guseva Canu I., Hopf N.B. (2020). Urinary 8-isoprostane as a biomarker for oxidative stress: A systematic review and meta-analysis. Toxicol. Lett..

[62] Sambiagio N., Sauvain J.-J., Berthet A., Auer R., Schoeni A., Hopf N.B. (2020). Rapid liquid chromatography-tandem mass spectrometry analysis of two urinary oxidative stress biomarkers: 8-oxodG and 8-isoprostane. Antioxidants.

[63] Cho Y.J., Park S.B., Park J.W., Oh S.R., Han M. (2018). Bisphenol A modulates inflammation and proliferation pathway in human endometrial stromal cells by inducing oxidative stress. Reprod. Toxicol..

[64] Watkins D.J., Ferguson K.K., Anzalota Del Toro L.V., Alshawabkeh A.N., Cordero J.F., Meeker J.D. (2015). Associations between urinary phenol and paraben concentrations and markers of oxidative stress and inflammation among pregnant women in Puerto Rico. Int. J. Hyg. Environ. Health.

[65] Sies H., Jones D.P. (2020). Reactive oxygen species (ROS) as pleiotropic physiological signalling agents. Nat. Rev. Mol. Cell Biol..

[66] Frijhoff J., Winyard P.G., Zarkovic N., Davies S.S., Stocker R., Cheng D., Knight A.R., Taylor E.L., Oettrich J., Ruskovska T. (2015). Clinical relevance of biomarkers of oxidative stress. Antioxid. Redox Signal..

[67] Ferguson K.K., McElrath T.F., Chen Y.H., Loch-Caruso R., Mukherjee B., Meeker J.D. (2015). Repeated measures of urinary oxidative stress biomarkers during pregnancy and preterm birth. Am. J. Obstet. Gynecol..

[68] Jarmund A.H., Giskeødegård G.F., Ryssdal M., Steinkjer B., Stokkeland L.M.T., Madssen T.S., Stafne S.N., Stridsklev S., Moholdt T., Heimstad R. (2021). Cytokine patterns in maternal serum from first trimester to term and beyond. Front. Immunol..

[69] Li L., Li D.S. (2021). Inter-individual variability and non-linear dose-response relationship in assessing human health impact from chemicals in LCA: Addressing uncertainties in exposure and toxicological susceptibility. Front. Sustain..

[70] Milenkovic D., Morand C., Cassidy A., Konic-Ristic A., Tomás-Barberán F., Ordovas J.M., Kroon P., De Caterina R., Rodriguez-Mateos A. (2017). Interindividual variability in biomarkers of cardiometabolic health after consumption of major plant-food bioactive compounds and the determinants involved. Adv. Nutr..

[71] Aleksandrova K., Koelman L., Rodrigues C.E. (2021). Dietary patterns and biomarkers of oxidative stress and inflammation: A systematic review of observational and intervention studies. Redox Biol..

[72] Furman D., Campisi J., Verdin E., Carrera-Bastos P., Targ S., Franceschi C., Ferrucci L., Gilroy D.W., Fasano A., Miller G.W. (2019). Chronic inflammation in the etiology of disease across the life span. Nat. Med..

[73] Piao X., Liu Z., Li Y., Yao D., Sun L., Wang B., Ma Y., Wang L., Zhang Y. (2019). Investigation of the effect for bisphenol A on oxidative stress in human hepatocytes and its interaction with catalase. Spectrochim. Acta A Mol. Biomol. Spectrosc..

[74] Aboul Ezz H.S., Khadrawy Y.A., Mourad I.M. (2015). The effect of bisphenol A on some oxidative stress parameters and acetylcholinesterase activity in the heart of male albino rats. Cytotechnology.

[75] Savastano S., Tarantino G., D’Esposito V., Passaretti F., Cabaro S., Liotti A., Liguoro D., Perruolo G., Ariemma F., Finelli C. (2015). Bisphenol-A plasma levels are related to inflammatory markers, visceral obesity and insulin-resistance: A cross-sectional study on adult male population. J. Transl. Med..

[76] Cai W., Yan Q., Deng Y., Guo Y. (2025). The correlation of bisphenol A exposure on inflammatory cytokines in preschool children. Cytokine.

[77] Wang K., Zhao Z., Ji W. (2019). Bisphenol A induces apoptosis, oxidative stress and inflammatory response in colon and liver of mice in a mitochondria-dependent manner. Biomed. Pharmacother..

[78] Wang K., Tang J., Shen D., Li Y., Nagaoka K., Li C. (2025). Bisphenol A exposure induces small intestine damage through oxidative stress, inflammation, and microbiota alteration in rats. Toxics.

[79] Lee Y., Baek J., Kwon Y. (2024). Assessing dietary bisphenol A exposure among Koreans: Comprehensive database construction and analysis using the Korea National Health and Nutrition Examination Survey. Food Addit. Contam. Part A.

[80] Quirós-Alcalá L., Eskenazi B., Bradman A., Ye X., Calafat A.M., Harley K. (2013). Determinants of urinary bisphenol A concentrations in Mexican/Mexican-American pregnant women. Environ. Int..

[81] Cui F.P., Yang P., Liu C., Chen P.P., Deng Y.L., Miao Y., Luo Q., Zhang M., Lu W.Q., Zeng Q. (2021). Urinary bisphenol A and its alternatives among pregnant women: Predictors and risk assessment. Sci. Total Environ..

[82] Völkel W., Colnot T., Csanády G.A., Filser J.G., Dekant W. (2002). Metabolism and kinetics of bisphenol A in humans at low doses following oral administration. Chem. Res. Toxicol..

[83] Lee A., Lim J.-E., Mok S., Kim S., Lee I., Moon H.-B., Choi K., Kim S., Park J. (2025). Exploring overlooked bisphenol exposure sources through a comprehensive 7-day investigation using time-activity diaries and urinary biomonitoring. Ecotoxicol. Environ. Saf..

[84] Jusko T.A., Shaw P.A., Snijder C.A., Pierik F.H., Koch H.M., Hauser R., Jaddoe V.W.V., Burdorf A., Hofman A., Tiemeier H. (2014). Reproducibility of urinary bisphenol A concentrations measured during pregnancy in the Generation R Study. J. Expo. Sci. Environ. Epidemiol..

[85] Fisher M., Arbuckle T.E., Mallick R., LeBlanc A., Hauser R., Feeley M., Koniecki D., Ramsay T., Provencher G., Bérubé R. (2015). Bisphenol A and phthalate metabolite urinary concentrations: Daily and across pregnancy variability. J. Expo. Sci. Environ. Epidemiol..

[86] LaKind J.S., Naiman D.Q. (2011). Daily intake of bisphenol A and potential sources of exposure: 2005–2006 National Health and Nutrition Examination Survey. J. Expo. Sci. Environ. Epidemiol..

[87] Zota A.R., Phillips C.A., Mitro S.D. (2016). Recent fast food consumption and bisphenol A and phthalates exposures among the U.S. population in NHANES, 2003–2010. Environ. Health Perspect..

[88] Sabry R.N., Moustafa R.S.I., Wahba S.A., Salah El-Din E.M., Boseila S., Youssef M.M., Abushady M.M., Hussein J., Medhat D., Morsy S.M. (2023). Influence of food consumption and packaging on urinary bisphenol-A level in a sample of Egyptian students. J. Arab. Soc. Med. Res. Arab..

[89] Beck A.L., Bräuner E.V., Uldbjerg C.S., Lim Y.-H., Boye H., Frederiksen H., Andersson A.-M., Jensen T.K. (2025). Maternal urinary concentrations of bisphenol A during pregnancy and birth size in children from the Odense Child Cohort. Environ. Health.

[90] Huang S., Li J., Xu S., Zhao H., Li Y., Zhou Y., Fang J., Liao J., Cai Z., Xia W. (2019). Bisphenol A and bisphenol S exposures during pregnancy and gestational age—A longitudinal study in China. Chemosphere.

[91] Sol C.M., van Zwol-Janssens C., Philips E.M., Asimakopoulos A.G., Martinez-Moral M.P., Kannan K., Jaddoe V.W.V., Trasande L., Santos S. (2021). Maternal bisphenol urine concentrations, fetal growth and adverse birth outcomes: A population-based prospective cohort. Environ. Health.

[92] Casas M., Valvi D., Ballesteros-Gómez A., Gascón M., Fernández M.F., García-Esteban R., Iñiguez C., Martínez D., Murcia M., Monfort N. (2016). Exposure to bisphenol A and phthalates during pregnancy and ultrasound measures of fetal growth in the INMA-Sabadell cohort. Environ. Health Perspect..

[93] Yang P., Lin B.G., Zhou B., Cao W.C., Chen P.P., Deng Y.L., Hou J., Sun S.Z., Zheng T.Z., Lu W.Q. (2021). Sex-specific associations of prenatal exposure to bisphenol A and its alternatives with fetal growth parameters and gestational age. Environ. Int..

[94] Hu C.Y., Li F.L., Hua X.G., Jiang W., Mao C., Zhang X.J. (2018). The association between prenatal bisphenol A exposure and birth weight: A meta-analysis. Reprod. Toxicol..

[95] Snijder C.A., Heederik D., Pierik F.H., Hofman A., Jaddoe V.W.V., Koch H.M., Longnecker M.P., Burdorf A. (2013). Fetal growth and prenatal exposure to bisphenol A: The Generation R Study. Environ. Health Perspect..

[96] Burstyn I., Martin J.W., Beesoon S., Bamforth F., Li Q., Yasui Y., Cherry N.M. (2013). Maternal exposure to bisphenol-A and fetal growth restriction: A case-referent study. Int. J. Environ. Res. Public Health.

[97] Cowell W., Jacobson M.H., Long S.E., Wang Y., Kahn L.G., Ghassabian A., Naidu M., Torshizi G.D., Afanasyeva Y., Liu M. (2023). Maternal urinary bisphenols and phthalates in relation to estimated fetal weight across mid to late pregnancy. Environ. Int..

[98] Braun J.M., Kalkbrenner A.E., Calafat A.M., Bernert J.T., Ye X., Silva M.J., Barr D.B., Sathyanarayana S., Lanphear B.P. (2011). Variability and predictors of urinary bisphenol A concentrations during pregnancy. Environ. Health Perspect..

[99] Strakovsky R.S., Schantz S.L. (2018). Impacts of bisphenol A (BPA) and phthalate exposures on epigenetic outcomes in the human placenta. Environ. Epigenet..

[100] Filardi T., Panimolle F., Lenzi A., Morano S. (2020). Bisphenol A and phthalates in diet: An emerging link with pregnancy complications. Nutrients.

[101] Chang C.H., Huang Y.F., Wang P.W., Lai C.H., Huang L.W., Chen H.C., Lin M.H., Yang W.N., Mao I.F., Chen M.L. (2019). Associations between prenatal exposure to bisphenol A and neonatal outcomes in a Taiwanese cohort study: Mediated through oxidative stress?. Chemosphere.

[102] Ekici M., Çakır Biçer N. (2025). National-level assessment of dietary bisphenol A exposure using EFSA-based models and health risks in Turkey. J. Food Compos. Anal..

[103] Amirkhizi F., Hamedi-Shahraki S., Rahimlou M. (2023). Dietary total antioxidant capacity is associated with lower disease severity and inflammatory and oxidative stress biomarkers in patients with knee osteoarthritis. J. Health Popul. Nutr..

[104] Peake C.G., Odgers-Jewell K., de Sousa C.J., English C.J., Ingabire A., Mayr H.L., Reidlinger D.P. (2025). Association between dietary inflammatory or oxidative stress indices and biomarkers in cardiometabolic and related conditions: A systematic literature review. Br. J. Nutr..

[105] Yeh K.L., Kautz A., Lohse B., Groth S.W. (2021). Associations between dietary patterns and inflammatory markers during pregnancy: A systematic review. Nutrients.

[106] Khalili Sadrabad E., Hashemi S.A., Nadjarzadeh A., Askari E., Akrami Mohajeri F., Ramroudi F. (2023). Bisphenol A release from food and beverage containers—A review. Food Sci. Nutr..

[107] Dodson R.E., Nishioka M., Standley L.J., Perovich L.J., Brody J.G., Rudel R.A. (2012). Endocrine disruptors and asthma-associated chemicals in consumer products. Environ. Health Perspect..

[108] Xue J., Liu W., Kannan K. (2017). Bisphenols, benzophenones, and bisphenol A diglycidyl ethers in textiles and infant clothing. Environ. Sci. Technol..

[109] Milne G.L., Dai Q., Roberts L.J. (2015). The isoprostanes—25 years later. Biochim. Biophys. Acta.

[110] Roberts L.J., Morrow J.D. (2000). Measurement of F_2_-isoprostanes as an index of oxidative stress in vivo. Free Radic. Biol. Med..

[111] Madsen M.T.B., Bjerregaard A.A., Furtado J.D., Halldorsson T.I., Strøm M., Granström C., Giovannucci E., Olsen S.F. (2019). Comparisons of estimated intakes and plasma concentrations of selected fatty acids in pregnancy. Nutrients.

