# Supplementary material for: Dietary Bisphenol A Exposure and Urinary Bisphenol A Levels During Late Pregnancy: Associations with Inflammatory and Oxidative Stress Biomarkers
Source: Nutrients. 2026 Aug 28;18(17):2836. doi: 10.3390/nu18172836 (PMC13567652; doi:10.3390/nu18172836)
Supplement: Supplementary file 1 [file nutrients-18-02836-s001.zip › Supplementary Table S1. Daily food group intake assessed by 3-day dietary records and FFQ.pdf]

Supplementary Table S1. Daily food group intake assessed by 3-day dietary records and FFQ

Food Groups and Items	3-Day Dietary Records Median (Q1– Q3)	FFQ Median (Q1– Q3)
**Milk and Dairy Products (g)**	208 (145 – 315)	300 (188.25 – 359.5)
Milk	22.5 (0 – 83.75)	43 (13 – 100)
Yogurt and Ayran	111.5 (45 – 180)	158 (78 – 219)
Cheese	40 (27.5 – 59.5)	44 (30 – 63.75)
Ice cream	0 (0 – 0)	2 (0 – 9)
Cream	0 (0 – 0)	0 (0 – 0)
**Meat and Meat Products (g)**	139.5 (114.25 – 178.75)	155.5 (126 – 198)
Red meat	34.5 (13.25 – 50.75)	19 (6.25 – 38)
White meat (poultry)	0 (0 – 26.5)	10.5 (3 – 21.75)
Processed meat	0 (0 – 0)	0 (0 – 2)
Seafood	0 (0 – 0)	5 (1.25 – 13.5)
Eggs	49 (26.25 – 60.25)	40 (26 – 51)
Legumes	13 (0 – 32.25)	13 (9 – 26)
Nuts and Seeds	16 (10 – 28.75)	46 (30.25 – 65)
**Vegetables and Fruits (g)**	469 (352.25 – 663.5)	822.03 (644.67 – 1027.85)
Vegetables	321 (226.75 – 421.5)	448.82 (323.36 – 579.84)
Fruits	146.5 (80.5 – 235.5)	332 (230 – 520.5)
**Bread and Cereals (g)**	202.5 (154 – 262.75)	148.5 (105.25 – 217)
Bread	78 (47.75 – 139.5)	76.5 (53.75 – 150)
Cereals	85.5 (61 – 122.75)	49 (31 – 64.75)
Processed cereals (cakes, crackers)	6 (0 – 48)	6.5 (2 – 15)
**Fats and Oils (g)**	45.5 (33.25 – 59.75)	33 (26.25 – 47.5)
Olive oil	10 (0 – 19)	5 (0 – 15)
Other vegetable oils	25 (9 – 33)	17 (10 – 25.75)
Solid fats	8.5 (0.25 – 15)	6.5 (3 – 13)
**Sweets (g)**	20.5 (6 – 39.5)	28 (16.25 – 47)
**Non-alcoholic beverages (g)**	200 (58 – 300)	376.75 (217.41 – 471.04)
**Other (sauces, canned foods, tomato paste) (g)**	6.5 (2 – 13.75)	14.44 (5.02 – 28.62)

Abbreviations: FFQ, Food Frequency Questionnaire
