# Supplementary material for: Dietary Bisphenol A Exposure and Urinary Bisphenol A Levels During Late Pregnancy: Associations with Inflammatory and Oxidative Stress Biomarkers
Source: Nutrients. 2026 Aug 28;18(17):2836. doi: 10.3390/nu18172836 (PMC13567652; doi:10.3390/nu18172836)
Supplement: Supplementary file 1 [file nutrients-18-02836-s001.zip › Supplementary Table S2. Descriptive statistics of maternal urinary BPA concentrations and oxidative stress and inflammatory biomarkers.pdf]

Supplementary Table S2. Descriptive statistics of maternal urinary BPA concentrations and oxidative stress and inflammatory biomarkers

Biomarkers	Median ($Q_1 - Q_3$)
**Urinary Biomarkers**	
BPA (μg/L)	209.9 (151.01 – 243.3)
Creatinine (mg/ml)	0.06 (0.04 – 0.1)
8-Isoprostane (ng/L)	676.01 (425.62 – 942.23)
8-OHdG (ng/ml)	85.37 (74.51 – 91.1)
BPA (μg/g creatinine)	3.19 (1.94 – 4.96)
8-Isoprostane (μg/g creatinine)	11.46 (5.74 – 20.9)
8-OHdG (μg/g creatinine)	1450.8 (878.02 – 2152.21)
**Serum Biomarkers**	
TAS (mmol/L)	1.48 (1.36 – 1.57)
TOS (μmol/L)	3.02 (2.71 – 3.73)
OSI	0.21 (0.18 – 0.25)
MDA (nmol/ml)	6.53 (5.93 – 7.58)
SOD (U/ml)	125.29 (89.39 – 191.17)
GPx (U/L)	407.06 (335.26 – 477.73)
CAT (U/ml)	31.95 (30.13 – 34.26)
CRP (mg/L)	2.69 (1.34 – 5.08)
IL-1β (pg/ml)	1581.99 (946.15 – 3429.41)
IL-6 (ng/L)	86.26 (64.05 – 152.9)
IL-10 (pg/ml)	181.67 (136.33 – 520.62)
TNF-α (ng/L)	146.95 (67.09 – 231.95)

Abbreviations: BPA, bisphenol A; TAS, total antioxidant status; TOS, total oxidant status; OSI, oxidative stress index; MDA, malondialdehyde; SOD, superoxide dismutase; GPx, glutathione peroxidase; CAT, catalase; CRP, C-reactive protein; IL-1β, interleukin-1 beta; IL-6, interleukin-6; IL-10, interleukin-10; TNF-α, tumor necrosis factor-alpha; 8-OHdG, 8-hydroxy-2′-deoxyguanosine
