# Supplementary material for: Dietary Bisphenol A Exposure and Urinary Bisphenol A Levels During Late Pregnancy: Associations with Inflammatory and Oxidative Stress Biomarkers
Source: Nutrients. 2026 Aug 28;18(17):2836. doi: 10.3390/nu18172836 (PMC13567652; doi:10.3390/nu18172836)
Supplement: Supplementary file 1 [file nutrients-18-02836-s001.zip › Supplementary Table S3. Associations between BPA-related dietary behaviors and creatinine-adjusted urinary BPA concentrations.pdf]

Supplementary Table S3. Associations between BPA-related dietary behaviors and creatinine-adjusted urinary BPA concentrations

Variable	Categories	n (%)	Median urinary BPA (µg/g creatinine)	*p*
Frequency of eating out	Never	5 (5.8)	2.80	0.711^{x}
	Every day	3 (3.5)	4.68	
	5–6 times/week	2 (2.3)	5.08	
	3–4 times/week	3 (3.5)	2.37	
	1–2 times/week	18 (20.9)	3.54	
	Once every 15 days	23 (26.7)	4.39	
	Once/month	32 (37.2)	2.86	
Frequency of fast-food consumption	Never	12 (14.0)	2.86	0.766^{x}
	Every day	2 (2.3)	2.89	
	3–4 times/week	2 (2.3)	8.06	
	1–2 times/week	13 (15.1)	3.33	
	Once every 15 days	23 (26.7)	3.33	
	Once/month	34 (39.5)	3.49	
Consumption of ready-to-eat packaged foods	Yes	27 (31.4)	3.05	0.113^{y}
	No	59 (68.6)	3.33	
Frequency of ready-to-eat packaged food consumption	Never	59 (68.6)	3.33	0.385^{x}
	3–4 times/week	2 (2.3)	2.92	
	1–2 times/week	7 (8.1)	3.33	
	Once every 15 days	9 (10.5)	3.74	
	Once/month	9 (10.5)	2.19	
Canned food consumption	Yes	46 (53.5)	3.69	0.945^{y}
	No	40 (46.5)	2.93	
Preferred canned food material	Metal can	28 (60.9)	3.55	0.643^{y}
	Glass jar	16 (34.8)	3.84	
	Other	2 (4.3)	-	

Abbreviations: BPA, bisphenol A. ^{x} Kruskal-Wallis H Test; ^{y} Mann-Whitney U Test. Categories with low observation counts were not included in the statistical comparison.
