# Supplementary material for: Dietary Bisphenol A Exposure and Urinary Bisphenol A Levels During Late Pregnancy: Associations with Inflammatory and Oxidative Stress Biomarkers
Source: Nutrients. 2026 Aug 28;18(17):2836. doi: 10.3390/nu18172836 (PMC13567652; doi:10.3390/nu18172836)
Supplement: Supplementary file 1 [file nutrients-18-02836-s001.zip › Supplementary Table S4. Correlations between estimated dietary BPA exposure and urinary BPA concentrations.pdf]

Supplementary Table S4. Correlations between estimated dietary BPA exposure and urinary BPA concentrations

**Variables**	**Urinary BPA (μg/L)**		**Creatinine-adjusted urinary BPA (μg/g creatinine)**	
	r	***p***	***r***	***p***
**Dietary BPA (3-day dietary records) (μg/kg/day)**	-0.009	0.932[x]	0.200	0.065[x]
**Dietary BPA (FFQ) (μg/kg/day)**	-0.062	0.574[x]	0.080	0.463[x]

Abbreviations: FFQ: Food Frequency Questionnaire; BPA: Bisphenol A. [x] Spearman's rho correlation
