# Supplementary material for: Dietary Bisphenol A Exposure and Urinary Bisphenol A Levels During Late Pregnancy: Associations with Inflammatory and Oxidative Stress Biomarkers
Source: Nutrients. 2026 Aug 28;18(17):2836. doi: 10.3390/nu18172836 (PMC13567652; doi:10.3390/nu18172836)
Supplement: Supplementary file 1 [file nutrients-18-02836-s001.zip › Supplementary Table S5. Correlations between maternal BPA exposure, oxidative stress, and inflammatory biomarkers.pdf]

Supplementary Table S5. Correlations between maternal BPA exposure, oxidative stress, and inflammatory biomarkers

	**Urinary BPA (μg/g creatinine)**		**Dietary BPA (3-day dietary records)**		**Dietary BPA (FFQ)**	
	r	***p***	***r***	***p***	***r***	***p***
**Urinary Biomarkers (μg/g creatinine)**						
8-Isoprostane	0.694	**<0.001**[x]	0.174	0.108[x]	0.139	0.203[x]
8-OHdG	0.880	**<0.001**[x]	0.198	0.067[x]	0.089	0.413[x]
**Serum Biomarkers**						
TAS	0.088	0.420[x]	0.016	0.888[x]	0.018	0.872[x]
TOS	-0.148	0.173[x]	0.147	0.177[x]	-0.004	0.974[x]
OSI	-0.156	0.151[x]	0.130	0.232[x]	-0.036	0.741[x]
MDA	-0.079	0.472[x]	0.038	0.730[x]	-0.034	0.760[x]
SOD	-0.045	0.682[x]	-0.015	0.893[x]	-0.116	0.289[x]
GPx	-0.025	0.819[x]	0.096	0.380[x]	0.092	0.401[x]
CAT	-0.155	0.155[x]	-0.091	0.406[x]	0.057	0.601[x]
CRP	0.038	0.730[x]	-0.049	0.656[x]	-0.127	0.243[x]
IL-1β	0.041	0.711[x]	0.175	0.107[x]	0.188	0.084[x]
IL-6	-0.011	0.918[x]	0.117	0.282[x]	0.114	0.297[x]
IL-10	-0.028	0.796[x]	0.221	**0.042**[x]	0.226	**0.037**[x]
TNF-α	-0.015	0.890[x]	0.216	**0.046**[x]	0.175	0.106[x]

Abbreviations: BPA, bisphenol A; FFQ, Food Frequency Questionnaire; TAS, total antioxidant status; TOS, total oxidant status; OSI, oxidative stress index; MDA, malondialdehyde; SOD, superoxide dismutase; GPx, glutathione peroxidase; CAT, catalase; CRP, C-reactive protein; IL-1β, interleukin-1 beta; IL-6, interleukin-6; IL-10, interleukin-10; TNF-α, tumor necrosis factor-alpha. [x] Spearman's rho correlation coefficients are presented. Urinary BPA and urinary biomarkers are expressed as creatinine-corrected values (μg/g creatinine). Dietary BPA values represent estimated intake based on 3-day dietary records and FFQ data.
