# Supplementary material for: Dietary Bisphenol A Exposure and Urinary Bisphenol A Levels During Late Pregnancy: Associations with Inflammatory and Oxidative Stress Biomarkers
Source: Nutrients. 2026 Aug 28;18(17):2836. doi: 10.3390/nu18172836 (PMC13567652; doi:10.3390/nu18172836)
Supplement: Supplementary file 1 [file nutrients-18-02836-s001.zip › Supplementary Table S6. Correlations of dietary total antioxidant capacity and dietary inflammatory index with BPA exposure, oxidative stress, and inflammatory.pdf]

Supplementary Table S6. Correlations of dietary total antioxidant capacity and dietary inflammatory index with BPA exposure, oxidative stress, and inflammatory biomarkers

	**Dietary total antioxidant capacity (mmol/1000 kcal)**		**Dietary inflammatory index**	
	r	***p***	***r***	***p***
**Estimated Dietary BPA Exposure**				
Dietary BPA (3-day dietary records)	-0.210	0.053[x]	-0.166	0.127[x]
Dietary BPA (FFQ)	-0.169	0.119[x]	-0.220	**0.042**[x]
**Urinary Biomarkers (µg/g creatinine)**				
BPA	0.189	0.081[x]	-0.232	**0.032**[x]
8-Isoprostane	0.015	0.893[x]	-0.180	0.097[x]
8-OHdG	0.161	0.139[x]	-0.260	**0.016**[x]
**Serum Biomarkers**				
TAS	-0.111	0.310[x]	-0.017	0.877[x]
TOS	-0.022	0.840[x]	-0.013	0.905[x]
OSI	-0.030	0.785[x]	0.033	0.761[x]
MDA	-0.073	0.502[x]	0.040	0.716[x]
SOD	0.112	0.305[x]	-0.107	0.328[x]
GPx	-0.129	0.236[x]	0.199	0.067[x]
CAT	-0.043	0.693[x]	0.029	0.794[x]
CRP	0.139	0.201[x]	-0.008	0.945[x]
IL-1β	-0.181	0.096[x]	0.029	0.794[x]
IL-6	-0.183	0.092[x]	0.101	0.356[x]
IL-10	-0.244	**0.024**[x]	0.131	0.227[x]
TNF-α	-0.293	**0.006**[x]	0.228	**0.035**[x]

Abbreviations: BPA, Bisphenol A; TAS, total antioxidant status; TOS, total oxidant status; OSI, oxidative stress index; MDA, malondialdehyde; SOD, superoxide dismutase; GPx, glutathione peroxidase; CAT, catalase; CRP, C-reactive protein; IL-1β, interleukin-1 beta; IL-6, interleukin-6; IL-10, interleukin-10; TNF-α, tumor necrosis factor-alpha; 8-OHdG, 8-hydroxy-2′-deoxyguanosine. [x] Spearman's rho correlation.
