# Supplementary material for: Dietary Bisphenol A Exposure and Urinary Bisphenol A Levels During Late Pregnancy: Associations with Inflammatory and Oxidative Stress Biomarkers
Source: Nutrients. 2026 Aug 28;18(17):2836. doi: 10.3390/nu18172836 (PMC13567652; doi:10.3390/nu18172836)
Supplement: Supplementary file 1 [file nutrients-18-02836-s001.zip › Supplementary Table S7. Indirect effects of oxidative stress and inflammatory biomarkers on the associations between maternal urinary BPA levels and fetal and .pdf]

Supplementary Table S7. Indirect effects of oxidative stress and inflammatory biomarkers on the associations between maternal urinary BPA levels and fetal and neonatal outcomes

Mediator	Head circumference<br>*β* (SE), *p*	Birth weight<br>*β* (SE), *p*	Birth length<br>*β* (SE), *p*	Estimated fetal weight<br>*β* (SE), *p*	Gestational age at birth<br>*β* (SE), *p*
**Urinary Biomarkers**					
8-Isoprostane	0.002 (0.026), 0.949	0.001 (0.022), 0.956	0.000 (0.019), 0.983	0.000 (0.009), 0.973	-0.001 (0.019), 0.977
8-OHdG	0.004 (0.029), 0.888	0.003 (0.023), 0.907	-0.007 (0.030), 0.829	0.006 (0.014), 0.671	-0.012 (0.037), 0.753
**Serum Biomarkers**					
TAS	0.003 (0.021), 0.879	0.012 (0.024), 0.612	0.006 (0.018), 0.729	0.005 (0.011), 0.648	0.004 (0.019), 0.830
TOS	-0.002 (0.027), 0.935	-0.001 (0.020), 0.978	-0.006 (0.026), 0.805	-0.003 (0.012), 0.774	0.005 (0.028), 0.872
MDA	-0.027 (0.042), 0.524	-0.031 (0.039), 0.435	-0.020 (0.031), 0.528	0.018 (0.021), 0.375	-0.021 (0.031), 0.492
SOD	0.004 (0.020), 0.835	0.011 (0.032), 0.738	0.008 (0.027), 0.757	-0.002 (0.010), 0.824	0.000 (0.021), 0.985
GPx	0.000 (0.013), 0.983	-0.001 (0.023), 0.973	-0.001 (0.018), 0.976	0.000 (0.009), 0.974	0.000 (0.015), 0.996
CAT	-0.015 (0.024), 0.534	0.001 (0.030), 0.979	-0.010 (0.026), 0.712	0.005 (0.012), 0.670	-0.020 (0.029), 0.492
CRP	0.006 (0.033), 0.864	0.006 (0.035), 0.854	0.002 (0.018), 0.923	0.001 (0.007), 0.921	0.001 (0.017), 0.950
IL-1β	0.016 (0.047), 0.738	-0.001 (0.030), 0.975	0.008 (0.040), 0.835	0.002 (0.012), 0.894	0.004 (0.037), 0.908
IL-6	0.000 (0.048), 0.995	0.000 (0.030), 0.999	0.000 (0.049), 0.995	0.000 (0.017), 0.996	0.000 (0.041), 0.998
IL-10	-0.001 (0.031), 0.984	0.004 (0.036), 0.903	0.012 (0.069), 0.865	-0.002 (0.019), 0.910	0.001 (0.037), 0.977
TNF-α	-0.003 (0.031), 0.911	-0.003 (0.027), 0.914	-0.006 (0.027), 0.836	0.000 (0.013), 0.997	-0.005 (0.030), 0.862

Abbreviations: BPA, bisphenol A; SE, standard error; TAS, total antioxidant status; TOS, total oxidant status; MDA, malondialdehyde; SOD, superoxide dismutase; GPx, glutathione peroxidase; CAT, catalase; CRP, C-reactive protein; IL-1β, interleukin-1 beta; IL-6, interleukin-6; IL-10, interleukin-10; TNF-α, tumor necrosis factor-alpha; 8-OHdG, 8-hydroxy-2′-deoxyguanosine. All models were adjusted for age, pre-pregnancy BMI, gestational age at birth, parity, infant sex, urinary creatinine, DII, and DTAC. For estimated fetal weight (EFW), gestational week at measurement was included instead of gestational age at birth, while all other covariates remained unchanged.
