# Supplementary material for: Dietary Bisphenol A Exposure and Urinary Bisphenol A Levels During Late Pregnancy: Associations with Inflammatory and Oxidative Stress Biomarkers
Source: Nutrients. 2026 Aug 28;18(17):2836. doi: 10.3390/nu18172836 (PMC13567652; doi:10.3390/nu18172836)
Supplement: Supplementary file 1 [file nutrients-18-02836-s001.zip › Supplementary Table S8. Indirect effects of oxidative stress and inflammatory biomarkers on the associations between maternal dietary BPA exposure and fetal an.pdf]

Supplementary Table S8. Indirect effects of oxidative stress and inflammatory biomarkers on the associations between maternal dietary BPA exposure and fetal and neonatal outcomes

Mediator	Head circumference β (SE), p	Birth weight β (SE), p	Birth length β (SE), p	Estimated fetal weight β (SE), p	Gestational age β (SE), p
**Urinary Biomarkers**					
8-Isoprostane	0.036 (0.051), 0.482	0.030 (0.047), 0.529	0.009 (0.043), 0.835	−0.003 (0.020), 0.878	−0.008 (0.043), 0.847
8-OHdG	−0.003 (0.034), 0.932	0.001 (0.025), 0.977	0.012 (0.031), 0.699	−0.008 (0.012), 0.522	0.020 (0.040), 0.607
**Serum Biomarkers**					
TAS	−0.002 (0.024), 0.945	−0.007 (0.030), 0.821	−0.004 (0.023), 0.868	−0.003 (0.012), 0.824	−0.002 (0.022), 0.908
TOS	0.002 (0.024), 0.947	0.001 (0.020), 0.964	0.003 (0.026), 0.913	0.002 (0.012), 0.861	−0.003 (0.027), 0.925
MDA	0.003 (0.030), 0.909	0.004 (0.029), 0.890	0.002 (0.020), 0.913	−0.002 (0.014), 0.903	0.002 (0.020), 0.908
SOD	−0.007 (0.024), 0.756	−0.018 (0.029), 0.533	−0.015 (0.028), 0.598	0.004 (0.011), 0.724	−0.001 (0.024), 0.959
GPx	−0.001 (0.016), 0.934	−0.005 (0.024), 0.840	−0.004 (0.023), 0.860	−0.002 (0.011), 0.862	0.000 (0.021), 0.986
CAT	0.005 (0.013), 0.679	0.002 (0.012), 0.876	0.002 (0.011), 0.838	0.000 (0.005), 0.976	0.005 (0.013), 0.698
CRP	0.032 (0.026), 0.218	0.036 (0.028), 0.202	0.010 (0.018), 0.578	0.004 (0.008), 0.583	0.006 (0.017), 0.728
IL-1β	-0.032 (0.045), 0.485	0.006 (0.040), 0.882	-0.018 (0.048), 0.715	-0.002 (0.016), 0.889	-0.008 (0.047), 0.858
IL-6	0.011 (0.044), 0.807	−0.005 (0.036), 0.888	−0.015 (0.054), 0.776	0.003 (0.017), 0.857	−0.006 (0.047), 0.907
IL-10	−0.003 (0.051), 0.957	0.032 (0.055), 0.562	0.082 (0.087), 0.348	−0.015 (0.028), 0.589	0.010 (0.058), 0.866
TNF-α	0.012 (0.048), 0.804	0.011 (0.040), 0.785	0.016 (0.043), 0.709	0.001 (0.020), 0.948	0.015 (0.045), 0.742

Abbreviations: BPA, bisphenol A; SE, standard error; 8-OHdG, 8-hydroxy-2′-deoxyguanosine; TAS, total antioxidant status; TOS, total oxidant status; MDA, malondialdehyde; SOD, superoxide dismutase; GPx, glutathione peroxidase; CAT, catalase; CRP, C-reactive protein; IL-1β, interleukin-1 beta; IL-6, interleukin-6; IL-10, interleukin-10; TNF-α, tumor necrosis factor-alpha. Models were adjusted for age, pre-pregnancy BMI, gestational age at birth, parity, infant sex, DII, and DTAC. Estimated fetal weight models included gestational age at measurement instead of gestational age at birth. Models including urinary biomarkers were additionally adjusted for urinary creatinine. Maternal dietary BPA exposure refers to BPA intake estimated from food frequency questionnaire (FFQ) data.
