# Supplementary material for: Dietary Bisphenol A Exposure and Urinary Bisphenol A Levels During Late Pregnancy: Associations with Inflammatory and Oxidative Stress Biomarkers
Source: Nutrients. 2026 Aug 28;18(17):2836. doi: 10.3390/nu18172836 (PMC13567652; doi:10.3390/nu18172836)
Supplement: Supplementary file 1 [file nutrients-18-02836-s001.zip › Supplementary Table S9. Adjusted associations between dietary fatty acid intakes assessed using the FFQ and urinary oxidative stress biomarkers.pdf]

Supplementary Table S9. Adjusted associations between dietary fatty acid intakes assessed using the FFQ and urinary oxidative stress biomarkers

Outcome variables	SFA β (95% CI)	*p*	MUFA β (95% CI)	*p*	PUFA β (95% CI)	*p*
8-Isoprostane	−0.16 (−0.49, 0.18)	0.354	−0.14 (−0.41, 0.14)	0.330	0.02 (−0.23, 0.26)	0.900
8-OHdG	−0.16 (−0.48, 0.15)	0.308	−0.03 (−0.29, 0.24)	0.848	−0.01 (−0.23, 0.22)	0.967

Abbreviations: FFQ, food frequency questionnaire; SFA, saturated fatty acids; CI, confidence interval; MUFA, monounsaturated fatty acids; PUFA, polyunsaturated fatty acids; 8-OHdG, 8-hydroxy-2′-deoxyguanosine. Separate multivariable linear regression models were constructed for SFA, MUFA, and PUFA intakes. All models were adjusted for age, gestational week, pre-pregnancy BMI, total energy intake, and urinary creatinine. Standardized β coefficients with 95% confidence intervals are presented.
